# Oxidative dehalogenation and denitration by a flavin-dependent monooxygenase is controlled by substrate deprotonation†
†Electronic supplementary information (ESI) available. See DOI: 10.1039/c8sc01482e


**DOI:** 10.1039/c8sc01482e

**Published:** 2018-08-08

**Authors:** Panu Pimviriyakul, Panida Surawatanawong, Pimchai Chaiyen

**Affiliations:** a School of Biomolecular Science and Engineering , Vidyasirimedhi Institute of Science and Technology (VISTEC) , Wangchan Valley , Rayong , 21210 , Thailand . Email: pimchai.chaiyen@vistec.ac.th; b Department of Biochemistry and Center for Excellence in Protein and Enzyme Technology , Faculty of Science , Mahidol University , Bangkok , 10400 , Thailand; c Department of Chemistry and Center of Excellence for Innovation in Chemistry , Faculty of Science , Mahidol University , Bangkok , 10400 , Thailand

## Abstract

Enzymes that are capable of detoxifying halogenated phenols (HPs) and nitrophenols (NPs) are valuable for bioremediation and waste biorefining. HadA monooxygenase was found to perform dual functions of oxidative dehalogenation (hydroxylation plus halide elimination) and denitration (hydroxylation plus nitro elimination). Rate constants associated with individual steps of HadA reactions with phenol, halogenated phenols and nitrophenols were measured using combined transient kinetic approaches of stopped-flow absorbance/fluorescence and rapid-quench flow techniques. Density functional theory was used to calculate the thermodynamic and electronic parameters associated with hydroxylation and group elimination steps. These parameters were correlated with the rate constants of hydroxylation, group elimination, and overall product formation to identify factors controlling individual steps. The results indicated that the hydroxylation rate constant is higher when the p*K*_a_ of the phenolic group is lower, *i.e.* it is more easily deprotonated, but not higher when the energy gap between the *E*_LUMO_ of the C4a-hydroperoxy-FAD intermediate and the *E*_HOMO_ of the phenolate substrate is lower. These data suggest that the substrate deprotonation has a higher energy barrier than the –OH transfer, and thus controls the hydroxylation step. For the group elimination, the process is controlled by the ability of the C–X bond to break. For the overall product formation (hydroxylation and group elimination combined), this analysis showed that the rate constant of product formation is dependent on the p*K*_a_ value of the substrate, indicating that the overall reaction is controlled by substrate deprotonation. This step also likely has the highest energy barrier and thus controls the overall process of oxidative dehalogenation and denitration by HadA. This report is the first to identify a key mechanistic factor controlling the enzymatic processes of oxidative dehalogenation and denitration.

## Abstract 2

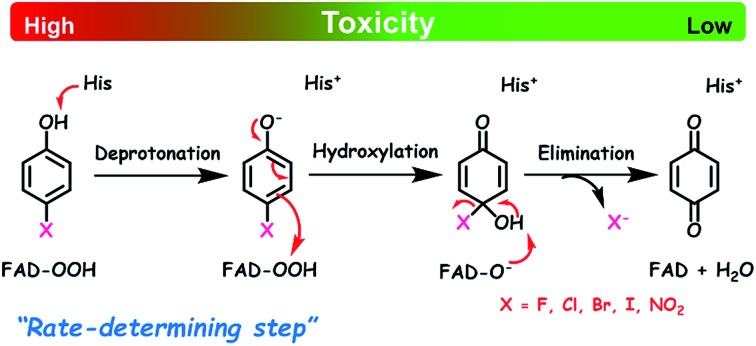
Enzymes that are capable of detoxifying halogenated phenols (HPs) and nitrophenols (NPs) are valuable for bioremediation and waste biorefining.

## Introduction

Halogenated phenols (HPs) and nitrophenols (NPs) such as 4-chlorophenol (4-CP), 4-nitrophenol (4-NP), 3,5-dibromo-4-hydroxybenzonitrile, chloronitroaromatic compounds, 2,4,6-trichlorophenol (2,4,6-TCP), and 2,4,5-trichlorophenol (2,4,5-TCP) are among the top agrochemicals widely used around the world as pesticides, disinfectant agents, wood preservatives and dyes.^1^–^3^ Due to their heavy usage and resistance to degradation, these compounds have been found to accumulate widely in soil and water.^1^,^4^,^5^ This creates serious concerns for human health, as these compounds are easily assimilated into the food chain and eventually make their way into the human body.^1^,^6^,^7^ HPs and NPs can cause severe health problems such as cancer, blood disorders, and organ damage, and can induce DNA damage and/or the formation of reactive oxygen species (ROS). Furthermore, a large intake of these compounds or a sudden exposure to them, which often occurs to poor workers in developing countries, can lead to acute death.^6^,^8^–^10^ Therefore, technologies that can detoxify HPs and NPs are necessary for environmental decontamination and subsequently human health.

**Scheme 1:**
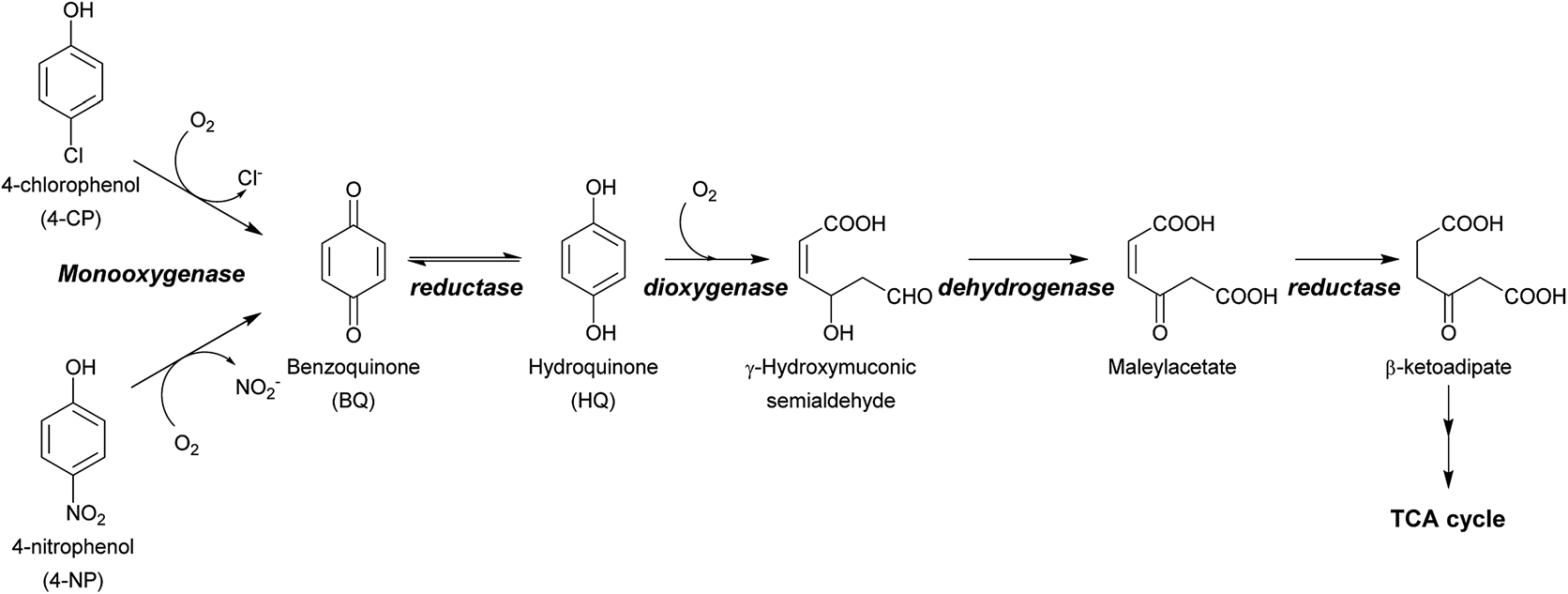
Dual functions of HadA in oxidative dehalogenation and denitration allow the single enzyme to detoxify halogenated phenols (HPs) and nitrophenols (NPs). The resulting hydroquinone can serve as a substrate for other enzymes in downstream steps which are commonly found in many soil microbes.^2^,^3^

A variety of technologies for degrading HPs and NPs have been developed. These include physical, chemical and biological processes. Although physical and chemical processes such as adsorption or advanced oxidation processes (AOPs) are efficient, they are costly, consume large amounts of energy, and also generate more toxic intermediates or byproducts after the initial treatment. Incineration of chlorophenol (CP) wastes is known to produce compounds with even greater toxicity, such as polychlorinated dibenzo-*p*-dioxins or polychlorinated dibenzofurans.^11^–^13^ On the other hand, biological degradation of HPs can be performed through efficient and clean processes. In addition to being sustainable and low-cost, detoxification *via* biodegradation using microbes or enzymes offers a means for converting unwanted toxic waste into value-added products. Such technology is very valuable for sustainable waste biorefining and promotes a circular economy where industries can both lower the cost of waste treatment and earn revenue through conversion of waste products into valuable compounds.^14^–^17^ The ultimate aim of waste biorefining is the engineering of super enzymes or engineered microbes for converting toxic waste into feedstock that can serve as carbon sources for other microbes for use in producing valuable products.^16^ To construct microbes that are capable of detoxifying HPs and NPs, enzymes that can remove halogen atoms (dehalogenation) or nitro groups (denitration) from aromatic rings are required. Dehalogenation or denitration is generally the rate-limiting step of HP and NP biodegradation because the resulting products with the halide or nitro groups removed can subsequently be metabolized by enzymes found in many species of soil bacteria, such as non-heme iron dioxygenases as well as other downstream enzymes further along the metabolic pathways^2^,^3^,^18^–^20^ (Scheme 1). Therefore, understanding the enzymatic mechanism controlling the dehalogenation of HPs and denitration of NPs is undoubtedly crucial for waste biorefining technology.

The enzymatic detoxification of HPs can occur *via* reductive or oxidative dehalogenation. Reductive dehalogenation does not require participation of oxygen and usually requires reducing equivalents in the forms of glutathione, reduced flavin or NAD(P)H. The reaction mechanisms of various types of reductive dehalogenases have been investigated in-depth.^21^–^25^ However, there is little mechanistic insight regarding enzymes catalyzing oxidative dehalogenation. Oxidative dehalogenases are usually encoded by operons found in aerobic bacteria such as the *tft* cluster from *Burkholderia cepacia* AC1100,^26^,^27^ the *tcp* cluster from *Ralstonia eutropha* JMP134,^28^,^29^ the *had* cluster from *Ralstonia pickettii* DTP0602,^30^–^33^ the *pcp* cluster from *Sphingobium chlorophenolicum*,^34^–^37^ and the *cph* cluster from *Arthrobacter chlorophenolicus* A6.^38^ These enzymes can dechlorinate CPs and incorporate hydroxyl groups. They are mostly single-component or two-component flavin-dependent monooxygenases. Dechlorination of pentachlorophenol (PCP) is catalyzed by the FAD-bound single-component pentachlorophenol hydroxylase (PcpB) which catalyzes both flavin reduction and substrate hydroxylation/dechlorination using a single polypeptide.^36^,^37^ Interestingly, most of the enzymes catalyzing oxidative dechlorination encoded in the operons mentioned above are two-component monooxygenases which perform two separate reactions, acting as a reductase and a dechlorinase. The reductase component generates reduced FAD (FADH^–^) to be used as a co-substrate by the dechlorinase in the dechlorination reaction. For these two-component flavin-dependent monooxygenases, the transfer of FADH^–^ from the reductase to the oxygenase does not require protein–protein interactions.^39^,^40^ Recently, the kinetics of a two-component flavin-dependent monooxygenase from *R. pickettii* DTP0602 (HadA) with 4-CP was investigated by transient kinetics. The results identified C4a-hydroperoxy-FAD and C4a-hydroxy-FAD as intermediates in the reaction.^33^

Most of the enzymes that catalyze the removal of a nitro group from NPs that have been reported to date are flavin-monooxygenases which catalyze oxidative denitration. Clusters that can degrade 4-nitrophenol (4-NP) and its derivatives include the *nph* and *nps* clusters from *Rhodococcus* sp. strain PN1,^41^–^43^ the *npc* cluster from *Rhodococcus opacus* SAO101,^44^ the *npd* cluster from *Arthrobacter* sp. Strain JS443,^45^ and the *pnp* cluster from *Pseudomonas* sp. strain WBC-3.^46^ Most of these enzymes have been studied only in crude lysates and have been characterized only for substrate utilization; however, none of these studies have performed detailed investigations into the reaction mechanisms. Interestingly, based on our previous work on HadA,^33^ we found that HadA shares a significant sequence similarity to flavin-dependent monooxygenases found in 4-NP degradation pathways such as NpcA from *R. opacus* SAO101 (42.5% identity),^44^ NpdA2 from *Arthrobacter* sp. Strain JS443 (42.4% identity),^45^ and NpsA1 from *Rhodococcus* sp. strain PN1 (42.5% identity).^43^ We speculated that HadA may also be able to catalyze hydroxylation plus nitro group elimination in addition to chloride elimination.

**Scheme 2:**
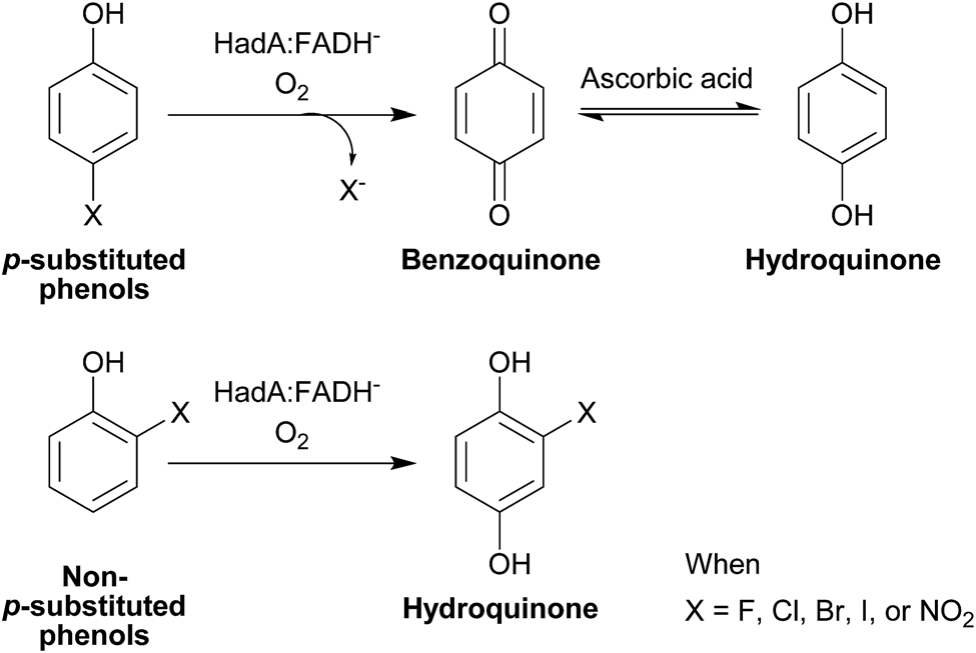
Reaction catalyzed by HadA. HadA catalyzes hydroxylation plus group elimination at position 4 of phenolic compounds with a *para*-substituent (upper scheme). For phenolic compounds without a substituent at the *para*-position (lower scheme), the enzyme only catalyzes hydroxylation.

If HadA contains dual catalytic functions of oxidative dehalogenation and denitration, the enzyme should be very valuable for waste biorefining, as this would allow the initial rate-limiting step of the detoxification pathways of both HPs and NPs to be catalyzed by a single enzyme to generate common metabolites that can be used by many enzymes commonly found in various soil microbes^2^,^3^ (Scheme 1). Our previous study has shown that HadA can catalyze dechlorination of various CPs but its reactivity towards other HPs and NPs is unknown.^33^ For the reaction of HadA with phenols containing a chloride substituent at position 4 (*para*-substituent), HadA catalyzes hydroxylation plus dechlorination at position 4 to generate benzoquinone. On the other hand, for phenols without the *para*-substituent, such as 2-chlorophenol (2-CP), the reaction catalyzed is merely hydroxylation to generate hydroquinone (HQ) derivatives (Scheme 2).

Based on the rationales explained above, we performed mechanistic characterization of the reaction of HadA. Substrate utilization of HadA was first explored to identify whether the enzyme can also use other HPs and NPs as substrates. The results indicated that HadA indeed performs dual functions of dehalogenation and denitration towards HPs and NPs, respectively. Rate constants associated with individual steps of HadA reactions with phenol, 4-fluorophenol (4-FP), 4-chlorophenol (4-CP), 4-bromophenol (4-BP), 4-iodophenol (4-IP), 4-nitrophenol (4-NP), 2-chlorophenol (2-CP), 2-iodophenol (2-IP), and 2-nitrophenol (2-NP) were explored using combined transient kinetic approaches of absorbance and fluorescence stopped-flow and rapid-quench flow techniques. Density functional theory was used to calculate thermodynamic parameters and electronic energies associated with the hydroxylation and group elimination steps. These parameters were correlated with the rate constants of hydroxylation, group elimination, and overall product formation to identify the factors controlling the individual steps based on quantitative structure–activity relationship (QSAR) analyses. The results indicated that the energy barrier associated with the hydroxylation step is higher than that of the group elimination step. Unlike other general flavin-dependent monooxygenases (hydroxylases), the hydroxylation catalyzed by HadA is unique in that the substrate deprotonation, not the hydroxyl group transfer, is the step that controls the overall product formation.

## Experimental section

### Preparation of HadA

HadA was purified according to a protocol previously described.^33^ Briefly, *hadA*-pET-11a was transformed into *E. coli* BL21 (DE3) cells and the cells were grown on an LB agar plate containing 50 μg mL^–1^ ampicillin. Cells were cultured in 3.8 L auto-induction medium containing 50 μg mL^–1^ ampicillin.^47^ The culture was incubated with shaking at 220 rpm and 37 °C. When the OD_600_ reached ∼1.0, the temperature was lowered to 25 °C and the culture was incubated with shaking overnight. A cell paste was harvested by centrifugation and kept at –80 °C until use. For purification, the cell paste was suspended with 50 mM sodium phosphate (NaPi) pH 7.0 containing 5 mM EDTA, 100 μM PMSF and 1 mM DTT. Cells were lysed by ultrasonication. Debris was removed by centrifugation at 27 000 × *g* for 1 h. Ammonium sulfate ((NH_4_)_2_SO_4_) precipitation (0–20%) was used to remove contaminant proteins. HadA was precipitated mostly in the 20–40% (NH_4_)_2_SO_4_ fraction which was further solubilized in 50 mM NaPi pH 6.5 containing 50 mM NaCl (buffer A). The dissolved HadA solution was dialyzed against 4 L of buffer A overnight and the resulting dialysate was loaded onto a DEAE-sepharose™ fast-flow (GE-healthcare) column (∼200 mL) which was pre-equilibrated with buffer A. Unbound proteins were removed with 5-column volumes of buffer A. Proteins were eluted with a linear gradient of 50–400 mM NaCl in 50 mM NaPi pH 6.5. Fractions of purified HadA were pooled and concentrated. The buffer was exchanged into 20 mM HEPES pH 7.5 by passing through a Sephadex™ G-25 (GE-healthcare) column. All purification processes were performed at 4 °C. The concentration of purified HadA was determined by measuring its absorbance at 280 nm (*ε*_280_ = 74.9 mM^–1^ cm^–1^) and the enzyme was stored at –80 °C.

### Single turnover reactions of HadA

Single turnover reactions of HadA with various substrates were investigated in order to compare the efficiency of product formation without complicating factors from reduced flavin regenerating enzymes.^33^ A reduced flavin solution was prepared by reducing an anaerobic solution of HadA (75 μM) and FAD (25 μM) in 20 mM HEPES pH 7.5 with a stoichiometric amount of sodium dithionite solution inside an anaerobic chamber. The completion of flavin reduction was monitored by a change in the flavin absorption spectra. The resulting HadA:FADH^–^ complex was mixed with solutions of air-saturated substrates (0.8 mM substrate and 0.13 mM oxygen) and ascorbic acid (1 mM) in closed microfuge tubes. The concentrations of reagents indicated are final concentrations after mixing. When the reactions were finished, protein was precipitated by adding an equal amount of acetonitrile. The protein precipitate was removed by centrifugation at 13 800 × *g* and 4 °C for 30 min. The supernatant was diluted 3-fold before being passed through an Amicon® Ultra-0.5 centrifugal filter device with a molecular weight cutoff of 10 kDa (Millipore). The filtrate was analyzed using an HPLC/diode array detector (DAD) or HPLC/DAD/mass spectroscopic detector (MSD) (Agilent Technologies) according to the protocol described below. As FADH^–^ was the limiting reagent, a coupling percentage value was calculated based on the amount of product formed or substrate consumed per amount of FADH^–^ used.

### Multiple turnover reactions of HadA

Reactions containing the substrate (100 μM), glucose-6-phosphate (G6P) (1 mM), glucose-6-phosphate dehydrogenase (G6PD) (0.5 unit per mL), C_1_ from *A. buamannii* (1 μM), NAD^+^ (5 μM), FAD (10 μM), HadA (10 μM) and ascorbic acid (1 mM) in 20 mM HEPES pH 7.5 were carried out in order to measure substrate conversion by HadA. Reactions were initiated by adding G6PD. At various time points, samples were drawn and quenched by adding an equal volume of acetonitrile. Samples were prepared by the same methods described above. The amount of substrate consumed and product formed at each time point was determined by HPLC/DAD.

### Product analysis

The products and substrates of the HadA reactions were analyzed according to a protocol previously reported^33^ using an HPLC (Agilent Technologies 1100 or 1260 Infinity series) equipped with a UV-visible diode array detector (DAD) for quantitative purposes and an electrospray ionization mass spectroscopic detector (ESI-MSD) (Agilent Technologies 6120 Quadrupole LC/MS) for identification of the products. Separation was done using a Nova-Pak® (Waters) C18 reverse phase column with a 4 μm particle size and a 3.9 × 150 mm column size as a stationary phase. A gradient of H_2_O/methanol containing 0.1% formic acid or a gradient of H_2_O/acetonitrile containing 0.1% formic acid was used as a mobile phase. The HPLC was run with a flow rate of 0.5 mL min^–1^ under ambient conditions. For DAD detection, wavelengths specific to particular substrates or products were employed for detection. For MSD detection, negative mode with a scan range of *m*/*z* from 10–500 or selected ion monitoring (SIM) mode for specific *m*/*z* was used. Quantitation of compounds was performed by comparing the signals to those of authentic compounds.

### Transient kinetics of the HadA reaction

Stopped-flow instruments, models SF-61SX and SF-61DX, equipped with a UV-visible spectrophotometer or fluorescence detector and a rapid-quench flow (RQF) apparatus model RQF-63 from TgK Scientific (Bradford-on-Avon, UK) were used to study the transient kinetics of the HadA reaction. Flow parts of instruments were made anaerobic by flushing with an anaerobic sodium dithionite solution overnight. Before performing experiments, excess sodium dithionite was rinsed out using anaerobic buffer at least three times. Typically, reactions were performed by mixing a solution of HadA : FADH^–^ (75 μM : 25 μM) binary complex with buffer solutions containing a phenolic substrate (0.8 mM) and various concentrations of oxygen (0.13, 0.31, and 0.61 mM) in 20 mM HEPES pH 7.5 at 25 °C using the stopped-flow apparatus. For UV-visible detection, absorbances at 380 nm and 450 nm were used to monitor the formation of C4a-hydroperoxy-FAD intermediates and oxidized FAD, respectively. For fluorescence detection, excitation wavelengths were set at 380 nm (Ex380) and 450 nm (Ex450) for detection of the C4a-hydroxy-FAD intermediate and oxidized FAD, respectively. An emission filter that can detect wavelengths >495 nm (*E*_m_ > 495) was used to detect both flavin species. The observed rate constants were analyzed using ProgramA software (developed by C. J. Chiu, R. Chang, J. Diverno and D. P. Ballou at the University of Michigan, Ann Arbor, MI, USA). RQF experiments were carried out inside an anaerobic glovebox. The conditions for RQF reactions were the same as those of the stopped-flow experiments except that only oxygen with a final concentration of 0.13 mM was used. The RQF machine allowed the reactions to be rapidly quenched at various time points from 0–100 s by adding an equal volume of 0.2 M HCl. Ascorbic acid (1 mM) was added to stabilize products in the hydroquinone form. Samples were collected, prepared, and analyzed by HPLC/DAD to measure the amount of product formed at various times. The plots of the amount of product formed *versus* reaction time were analyzed with a single exponential equation using KaleidaGraph software (Synergy Software) to obtain the rate constants of product formation.

### Density functional theory (DFT) calculations

All molecules were drawn using Arguslab software (developed by M. Thompson, Seattle, WA, USA). The FAD was truncated to include the isoalloxazine ring up to the C_α_ and C_β_ positions. All calculations were performed using the Guassion 09 program.^48^ All geometries were optimized in the gas phase using the B3LYP/6-31G(d) basis set.^49^–^55^ Frequency calculation was performed to ensure that there is no imaginary frequency for the minima and to obtain the corrections to the thermodynamic quantities. The basis set 6-311+G(d,p)^52^–^54^ was used for calculations using the single point energy with a conductor-like polarizable continuum model (CPCM)^56^ and UAKS radii^56^ in H_2_O solvent to obtain the solvent corrected Gibbs free energy (Δ*G*), highest occupied molecular orbital energy (*E*_HOMO_), and lowest unoccupied molecular orbital energy (*E*_LUMO_) values of the compounds. This method has been successfully used to study the reaction mechanisms of flavoenzyme reactions.^57^,^58^

## Results

### Identification of halogenated- and nitro-phenolic substrates of HadA

**Chart 1:**
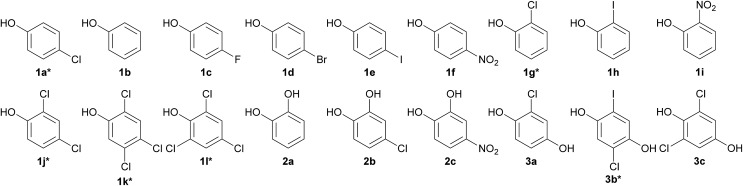
Structures of HadA substrates identified by multiple turnover reactions. Group 1: phenol derivatives: **1a** 4-chlorophenol (4-CP)*, **1b** phenol, **1c** 4-fluorophenol (4-FP), **1d** 4-bromophenol (4-BP), **1e** 4-iodophenol (4-IP), **1f** 4-nitrophenol (4-NP), **1g** 2-chlorophenol (2-CP), **1h** 2-iodophenol (2-IP), **1i** 2-nitrophenol (2-NP), **1j** 2,4-dichlorophenol (2,4-DCP), **1k** 2,4,5-trichlorophenol (2,4,5-TCP), and **1l** 2,4,6-trichlorophenol (2,4,6-TCP). Group 2: catechol derivatives: **2a** catechol, **2b** 4-chlorocatechol (4-CC), and **2c** 4-nitrocatechol (4-NC). Group 3: hydroquinone derivatives: **3a** chlorohydroquinone (CHQ), **3b** 2,5-dichlorohydroquinone (2,5-DCHQ), and **3c** 2,6-dichlorohydroquinone (2,6-DCHQ). * refers to compounds that were previously identified as substrates for HadA.

Previous studies have shown that HadA can use CPs (4-CP, 2-CP, 2,4-DCP, 2,4,5-TCP, 2,4,6-TCP and 2,5-DCHQ) as substrates^31^,^33^ but it was not known whether the enzyme can dehalogenate other HPs. Based on amino acid sequence analysis, HadA also has the potential to use NPs as substrates. In order to explore whether HadA can use HPs and NPs as substrates, multiple turnover reactions of HadA with HPs and NPs were performed, and the reactions were monitored by absorption spectroscopy. Bathochromic shifts of substrate absorption to longer wavelengths were used to initially identify whether the compounds can serve as substrates for HadA and whether hydroxyl groups were incorporated. Bathochromic shifts were observed for the following HPs and NPs (these compounds have never been reported as substrates for HadA): 4-FP, 4-BP, 4-IP, 4-NP, 2-IP, and 2-NP (data not shown). HadA can also use catechol derivatives (catechol, 4-CC, and 4-NC) and hydroquinone derivatives (CHQ and 2,6-DCHQ) as substrates. The structures of HPs and NPs that can serve as substrates for HadA are shown in Chart 1.

Products from the single turnover reactions of HadA and potential HP and NP substrates identified above were analyzed using HPLC/DAD/MSD. The MSD profiles of the products were compared with standard compounds. According to Scheme 2, the results show that HadA can convert all *para*-substituted phenols (4-FP, 4-BP, 4-IP, and 4-NP) to the same product, hydroquinone (HQ), *via* hydroxylation plus group elimination (Fig. S1†). For phenols without a substituent at the *para*- position (phenol, 2-IP, and 2-NP), the compounds were hydroxylated at position 4 without group elimination to generate HQ, iodohydroquinone (IHQ), and nitrohydroquinone (NHQ), respectively (Fig. S1 and S2†).

Analysis of the percentage of product formation or substrate consumption from single turnover reactions revealed that most of the substrates with substituents at the *para*- and *ortho*-positions have coupling percentages of around 50–60%, while phenol has a coupling ratio of only ∼30% (Table 1). These values imply that substrates with a mono-substituent bind to HadA with a similar reaction geometry so that hydroxyl group transfer from the flavin intermediate to the substrates occurs with similar efficiency. All of these data suggest that HadA is not a simple dechlorinase, but is a monooxygenase that can catalyze hydroxylation plus group elimination (dehalogenation and denitration) of a broad range of HPs and NPs.

**Table 1:** Product identification and percentages of product formation (% coupling) from single turnover reactions and percentages of conversion (% conversion) from multiple turnover reactions of HadA with HPs and NPs

Substrate	Product^*a*^	% coupling	% conversion^*b*^
Phenol	HQ	32 ± 2	75
4-FP	HQ	50 ± 1	88
4-CP^*d*^	HQ	49 ± 4	100
4-BP	HQ	51 ± 1	100
4-IP	HQ	55 ± 2	100
4-NP	HQ	53 ± 1	100
2-CP^*d*^	CHQ	43 ± 7^*c*^	100
2-IP	IHQ	50 ± 14^*c*^	100
2-NP	NHQ	55 ± 11^*c*^	96

^*a*^Product was characterized in the presence of ascorbic acid.

^*b*^Conversion percentages were calculated from multiple turnover reactions at the time points where the amount of substrate or product was not changed (2–10 h).

^*c*^Product formation from single turnover reactions was estimated based on the depletion of the substrate.

^*d*^Data obtained from a previous report.^33^

### Use of HadA to catalyze group elimination of HPs and NPs

In order to investigate whether HadA can catalyze hydroxylation/group elimination of HPs and NPs in a continuous process, multiple turnover reactions of HadA with various substrates (100 μM) were carried out to measure the overall rates of biodetoxification. Samples were collected and quenched at various time points over a period of 2–10 h, and analyzed by HPLC/DAD. The data showed that HadA could completely convert most single substrates into the product (100%) within 2 h (Fig. S3†). The percentages of conversion of all substrates were calculated and the results are summarized in Table 1. Most of the substrates were completely depleted, indicating that HadA has strong potential for use as a biocatalyst for detoxifying many HPs and NPs.

**Fig. 1:**
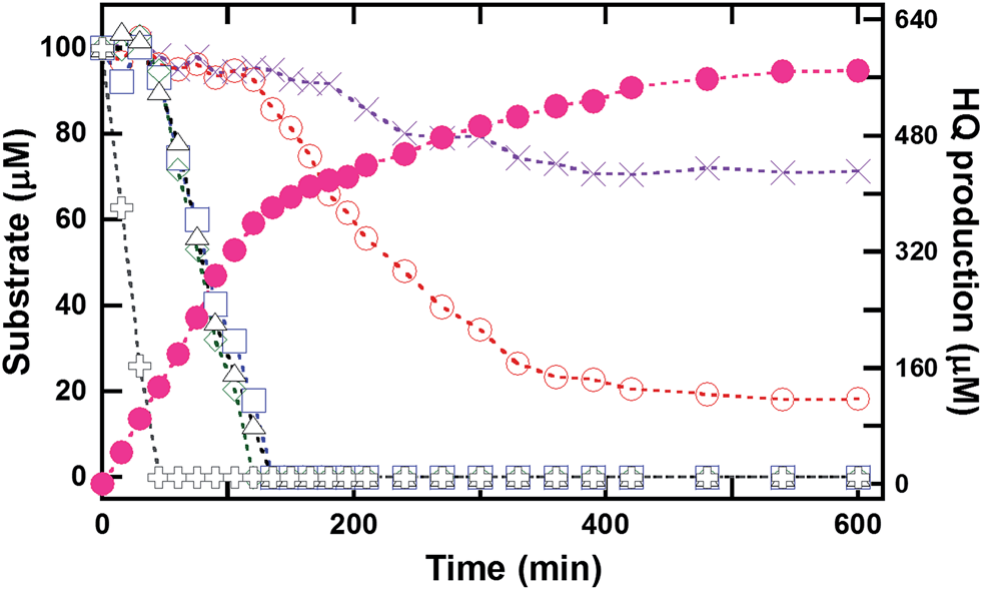
Multiple turnover reactions of HadA with a mixture of *para*-substituted phenols. Multiple turnover reactions of HadA with a mixture of six substrates (100 μM each) including phenol (purple cross), 4-FP (empty red circle), 4-CP (empty blue square), 4-BP (empty green diamond), 4-IP (empty black triangle), and 4-NP (black plus) were carried out, and the amounts of products formed and substrates remaining were analyzed as a function of time. The amount of the product (HQ) formed is indicated by filled magenta circles.

As a practical detoxification process would require a biocatalyst that works efficiently with a mixture of substrates, the reaction of HadA with a mixture of HPs or CPs was also investigated. Multiple turnover reactions (12 mL) of HadA with a mixture of 4-FP, 4-CP, 4-BP, 4-IP, 4-NP and phenol (100 μM each) were analyzed for substrate consumption and product formation over time. Samples were taken from the reaction over the course of 10 h. The results in Fig. 1 show that 4-NP is the first substrate to be consumed (within 45 min), followed by 4-CP, 4-BP, and 4-IP with comparable rates, while 4-FP was the slowest substrate to be consumed among all of the HPs utilized. Phenol was the slowest substrate among all the substrates explored, and it could not be completely hydroxylated even after 10 h. Product analysis indicated that HQ was the only product detected for all of these reactions, in agreement with the results shown in the previous section (Table 1). The amount of substrate consumed was consistent with the amount of product generated. Unlike multiple turnover reactions of individual substrates (Fig. S3†), the rates of consumption of individual HPs and NPs within the compound mixture are different, indicating that HadA has a preference towards different HPs and NPs in the order 4-NP > 4-IP ≥ 4-BP ≥ 4-CP > 4-FP > phenol.

### Investigation of the HadA mechanism using transient kinetics

**Scheme 3:**
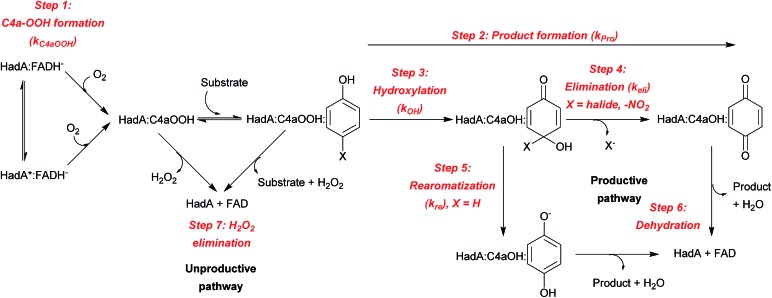
Kinetic scheme and reaction steps associated with HadA reaction.

**Fig. 2:**
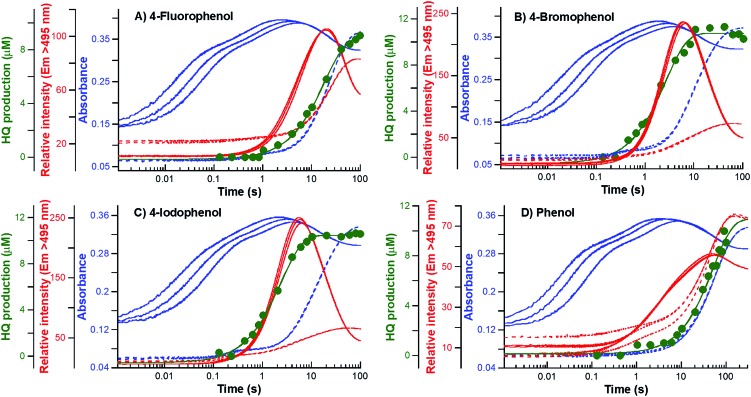
Kinetic traces of the reaction of HadA:FADH^–^ with oxygen in the presence of 4-FP, 4-BP, 4-IP, and phenol. Kinetic traces obtained from single turnover reactions of a HadA:FADH^–^ binary complex (75 μM and 25 μM, respectively) with oxygenated (A) 4-FP, (B) 4-BP, (C) 4-IP, and (D) phenol (0.8 mM substrate with 0.13, 0.31, and 0.61 mM oxygen) in 20 mM HEPES pH 7.5 at 25 °C. Blue lines are kinetic traces detected by stopped-flow spectrophotometry at wavelengths of 380 (solid line) and 450 nm (dashed line). Red lines are fluorescence signals detected by stopped-flow fluorescence using the excitation wavelengths of 380 nm (solid line) and 450 nm (dashed line) with emission wavelengths >495 nm. The green line is a calculated exponential curve that is correlated with the kinetics of product formation analyzed by rapid-quench flow (RQF) and HPLC/DAD techniques.

The results from the previous sections suggest that although HadA can hydroxylate/eliminate substituents from HPs/NPs with similar yields (Table 1), the enzyme's reactivity towards various substrates is different (Fig. 1). In a previous study, the kinetic scheme of the reaction of HadA:FADH^–^ with 4-CP was obtained from transient kinetic studies using stopped-flow spectrophotometry/fluorescence and rapid-quench flow techniques and is summarized in Scheme 3.^33^ In order to investigate the factors that control the reactivity of HadA and better understand the reaction mechanism, we used similar approaches but the reactions were carried out with various substrates including phenol, 4-FP, 4-BP, 4-IP, 4-NP, 2-CP, 2-IP, and 2-NP to measure the rate constants associated with the individual steps including C4a-hydroperoxy-FAD formation (*k*_C4aOOH_), product formation (*k*_Pro_), hydroxylation (*k*_OH_), and halide or nitro group elimination (*k*_Eli_). The results obtained from the transient kinetic experiments are described below. Only the data of 4-FP, 4-BP, 4-IP, and phenol are shown in Fig. 2. The kinetic traces for the reactions of the other substrates are found in Fig. S4.†

#### A. C4a-hydroperoxy-FAD formation step

Stopped-flow experiments with absorption detection were carried out to measure the rate constants associated with the conversion of HadA:FADH^–^ first to the C4a-hydroperoxy-FAD intermediate which has a maximum absorbance peak at around 380 nm (HadA:C4aOOH in Scheme 3) and then to oxidized FAD which has a maximum absorbance peak at around 450 nm (FAD in Scheme 3). Accordingly, the reactions of HadA:FADH^–^ with oxygen in the presence of all the substrates were monitored at 380 and 450 nm except those of 4-NP and 2-NP, for which the absorption changes could not be monitored due to interference from the strong absorbance of the –NO_2_ substituent. Kinetic traces obtained from the reactions in the presence of 4-FP, 4-BP, 4-IP, phenol (blue lines in Fig. 2A–D), 2-CP, and 2-IP (blue lines in Fig. S4B and C†) showed three observable phases. The first (0.002–0.2 s) and second (0.2–2 s) phases showed a large increase of absorbance at 380 nm (solid blue lines) and a negligible change in absorbance at 450 nm (dashed blue lines). The kinetics of these two phases were similar for all of the substrate reactions (Fig. S5A†), implying that these steps are not involved in substrate binding or transformation. These two steps were interpreted as the reactions of slow and fast reacting HadA:FADH^–^ complexes (represented as HadA:FADH^–^ and HadA*:FADH^–^ species in Scheme 3) with oxygen to form C4a-hydroperoxy-FAD before the substrate binding (step 1 in Scheme 3).^33^ The plots of the observed rate constants of these two phases for the reactions of all of the substrates also displayed linear dependency on oxygen concentration (data not shown), and yielded bimolecular rate constants of C4a-hydroperoxy-FAD formation (*k*_C4aOOH_) within a similar range. The values were also similar to those from the reaction performed in the absence of the substrates (Table 2). The substrate-independent formation of C4a-hydroperoxy-FAD strongly supports the view that the first two steps of the reaction are only involved in C4a-hydroperoxy-FAD formation and the substrate binds after the intermediate forms (Scheme 3).

**Table 2:** Rate constants associated with individual steps of reactions of the HadA:FADH^–^ binary complex with oxygen and various phenolic substrates obtained from transient kinetic analysis

Substrate	Rate constant of C4a-hydroperoxy-FAD formation (*k*_C4aOOH_)		Rate constants of individual steps (s^–1^)				
	1^st^ phase (M^–1^ s^–1^)	2^nd^ phase (M^–1^ s^–1^)	Product formation (*k*_Pro_)	Hydroxylation (*k*_OH_)	Group elimination (*k*_Eli_)	Rearo-matization (*k*_Re_)	Dehydration (*k*_De_)
Phenol	7.4 ± 0.5 × 10^4^	2.7 ± 0.2 × 10^3^	0.007 ± 0.001	0.013 ± 0.002	—^*c*^	0.014 ± 0.001	0.004 ± 0.001
4-FP	8.8 ± 0.6 × 10^4^	3.4 ± 0.3 × 10^3^	0.027 ± 0.001	0.06 ± 0.01	0.05 ± 0.01	—	0.014 ± 0.002
4-CP^*a*^	7.0 ± 0.2 × 10^4^	3.8 ± 0.3 × 10^3^	0.12 ± 0.01	0.18 ± 0.02	0.37 ± 0.01	—	0.029 ± 0.002
4-BP	7.9 ± 0.3 × 10^4^	4.1 ± 0.3 × 10^3^	0.20 ± 0.02	0.30 ± 0.03	0.66 ± 0.02	—	0.028 ± 0.001
4-IP	6.9 ± 0.4 × 10^4^	3.6 ± 0.2 × 10^3^	0.23 ± 0.01	0.33 ± 0.04	0.76 ± 0.01	—	0.028 ± 0.001
4-NP	ND^*b*^	ND	1.78 ± 0.16	ND	ND	—	ND
2-CP	10.7 ± 0.2 × 10^4^	4.4 ± 0.4 × 10^3^	0.10 ± 0.02	0.20 ± 0.04	—	0.20 ± 0.02	0.05 ± 0.01
2-IP	9.5 ± 0.4 × 10^4^	3.8 ± 0.5 × 10^3^	0.14 ± 0.04	0.24 ± 0.07	—	0.32 ± 0.04	0.06 ± 0.02
2-NP	ND	ND	0.10 ± 0.02	0.21 ± 0.05	—	0.19 ± 0.02	0.05 ± 0.01
None^*a*^	6.4 ± 0.4 × 10^4^	3.3 ± 0.5 × 10^3^	—	—	—	—	—

^*a*^Data from ref. 33.

^*b*^ND indicates rate constants that cannot be determined.

^*c*^A dash (—) represents a case in which the reaction does not occur.

#### B. Hydroxylation (C4a-hydroxy-FAD formation) step

As the HadA-bound C4a-hydroxy-FAD intermediate (HadA:C4aOH in Scheme 3) is highly fluorescent, while C4a-hydroperoxy-FAD is not,^33^ the rate constant associated with the hydroxylation step (*k*_OH_) can be measured by monitoring the fluorescence increase due to formation of C4a-hydroxy-FAD. Stopped-flow experiments similar to those carried out in Section A were performed and monitored by fluorescence using excitation wavelengths of 380 nm (Ex380, to detect C4a-hydroxy-FAD) and 450 nm (Ex450, to detect oxidized FAD) with emission wavelengths >495 (*E*_m_ > 495 nm). Fluorescence kinetic traces of the reactions in the presence of each substrate (4-FP, 4-BP, 4-IP, phenol, 4-NP, 2-CP, 2-IP, and 2-NP) are shown as red lines in Fig. 2 and S4.† All reactions showed three observable phases except for 4-NP, which had a very low change in relative intensity due to signal interference from the substrate. The first phase (0.002–0.2 s) is a lag phase with a negligible change of fluorescence at both excitation wavelengths. This phase involves the formation of HadA:C4a-hydroperoxy-FAD as its kinetics are concomitant with signals detected by stopped-flow in the absorption mode (described in Section A). The second phase (0.2–5 s) showed a large fluorescence increase at Ex380 (solid red lines) and a small fluorescence increase at Ex450 (dashed red lines). This phase represents the formation of C4a-hydroxy-FAD as the compound is highly fluorescent. This step is the hydroxylation step in which an –OH group is transferred from C4a-hydroperoxy-FAD to a substrate, resulting in HadA:C4a-hydroxy-FAD formation (step 3, Scheme 3). The rate constants of this phase (*k*_OH_) were independent of oxygen concentration in all substrate reactions, which is in agreement with our interpretation that this step does not involve the reaction with molecular oxygen (Scheme 3). The values of hydroxylation rate constants (*k*_OH_) obtained from these data are found to vary among the different substrates (Table 2 and Fig. S5B†).

The last phase (5–100 s) showed a large decrease of fluorescence at Ex380 and an increase in fluorescence at Ex450, implying that this phase represents the formation of oxidized FAD, in agreement with the data obtained by stopped-flow with absorption detection (dashed blue traces in Fig. 2, and S4B and C†). This step is a combination of the dehydration reaction of HadA:C4a-hydroxy-FAD and H_2_O_2_ elimination from HadA:C4a-hydroperoxy-FAD in the uncoupling pathway (steps 6 and 7, Scheme 3). Analysis of the individual rate constants associated with group elimination and the following steps requires the results from rapid-quench flow experiments as described in Section C.

#### C. Product formation and group elimination steps

The rate constants of product formation were determined using rapid-quench flow (RQF) techniques in combination with HPLC/DAD analysis. The kinetic traces obtained from the RQF experiments of the reactions of HadA:FADH^–^ with various phenolic substrates were analyzed using a single exponential equation to obtain the rate constant of product formation (*k*_Pro_). The results (filled green circular lines in Fig. 2 and S4†) show that the rate of product formation (combined steps of hydroxylation and group elimination or re-aromatization, step 2, Scheme 3) is slower than the rate of hydroxylation alone (measured from fluorescence stopped-flow experiments, red lines, Fig. 2), indicating that hydroxylation and group elimination or re-aromatization steps exist as discrete steps. The results also show that the rates of product formation vary among the different substrates (Fig. S5C†). Based on the overall reaction of HadA (step 2, Scheme 3), for *para*-substituted phenols (4-FP, 4-CP, 4-BP, 4-IP, and 4-NP), the rate constants measured from RQF (*k*_Pro_) are associated with two consecutive steps of hydroxylation (*k*_OH_) and group elimination (*k*_Eli_) (steps 3 and 4, Scheme 3) while for the reactions of *ortho*-substituted phenols, the RQF rate constants are associated with two consecutive steps of hydroxylation (*k*_OH_) and re-aromatization (*k*_re_) (steps 3 and 5, Scheme 3).

As the reaction is composed of bifurcated paths of coupling and uncoupling pathways, the rate constants associated with each path could be calculated from the percentage of product formation (eqn (1) and (2)).

1 *k*_obs_ = *k*_coupling_ + *k*_uncoupling_

2
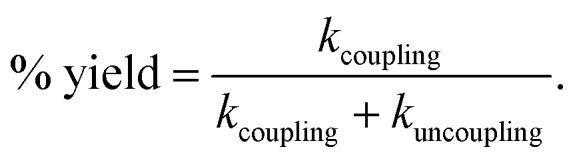



Individual values of rate constants were calculated based on eqn (3) or eqn (4) and are summarized in Table 2.3
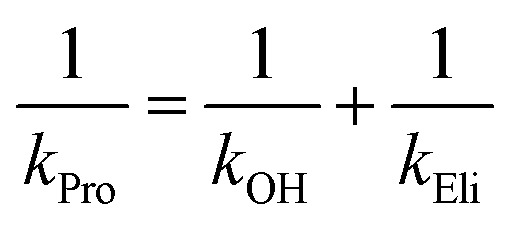

4
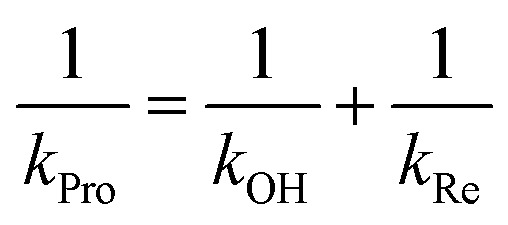



In most of the reactions, the kinetics of product formation (filled green circles) are faster than the kinetics of the appearance of the final oxidized FAD species (final phase of blue lines). Exceptions were observed for the reaction of 4-FP and phenol in which the observed rate constants of product formation and oxidized FAD formation are in the same range. These results suggest that for most substrates product formation is significantly faster than the final flavin dehydration step (step 6, Scheme 3) except for the reactions of 4-FP and phenol in which product formation was much slower than for the other substrates.

**Scheme 4:**
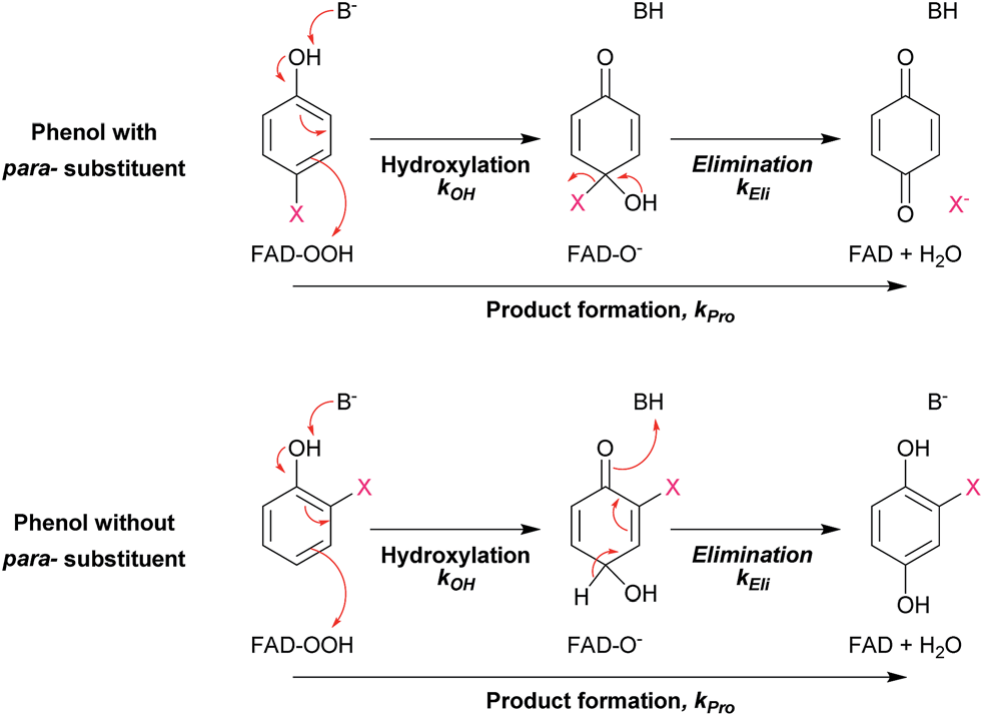
Elementary steps involved in product formation in the HadA reaction. The upper scheme shows the reaction of HadA and phenols with a *para*-substituent resulting in the formation of quinone *via* hydroxylation and group elimination. The lower scheme shows the reaction of HadA and phenols without a *para*-substituent resulting in the formation of hydroquinone *via* hydroxylation and re-aromatization. FAD-OOH, FAD-O^–^, and FAD represent C4a-hydroperoxy-FAD, C4a-hydroxide-FAD, and oxidized FAD, respectively.

The results in Table 2 also indicate that for most of the substrates, particularly the *para*-substituted phenol, the hydroxylation rate constants are lower than the group elimination rate constants, indicating that the hydroxylation step has a higher energy barrier than the group elimination step. As the overall product formation consists of (i) hydroxylation and (ii) group elimination or re-aromatization, individual elementary steps involved in the reaction of HadA with *para*-substituted and *ortho*-substituted phenols can be described as in Scheme 4.

### Quantitative structure–activity relationship (QSAR) analysis to identify factors controlling the individual steps of the HadA reaction

The kinetic results in the previous section (Table 2 and Fig. 2 and S4†) yielded valuable information of rate constants associated with product formation, hydroxylation and group-elimination steps. These results indicated that the substrate binds after C4a-hydroperoxy-FAD forms. Different rate constants of product formation, C4a-hydroxy-FAD formation (hydroxylation step) and group elimination were also obtained for the reactions performed with various substrates (Table 2 and Fig. S5†). In order to keep the QSAR parameters consistent, substrates subjected to the same substituent effects were compared. The rate constants of hydroxylation, group elimination and overall product formation of *para*-substituted phenols (4-FP, 4-CP, 4-BP, 4-IP, and 4-NP) and phenol were used in the QSAR analysis below since the inductive and steric effects the *ortho*-substituted phenols are subjected to are different from those for *para*-substituted phenols.

The hydroxylation step consists of deprotonation of the phenolic substrate and –OH transfer from the C4a-hydroperoxy-FAD intermediate to the C4 of phenol (Scheme 4). From related studies, these two processes were proposed to proceed either in a concerted^59^ or in a step-wise manner that requires substrate activation before –OH transfer.^60^–^62^ To gain insights into the effects of the deprotonation and the –OH transfer on the overall hydroxylation step, we discuss these two processes separately. For dehalogenation and denitration reactions, the hydroxylated substrate eliminates a *para*-substituent to produce a benzoquinone product. The calculated electronic and thermodynamic parameters including the experimental physico-chemical parameters associated with the individual steps were used for correlation with the experimental rate constants to establish the QSAR and to identify factors promoting higher reaction rates. Although the electronic and thermodynamic energies associated with the HadA structure could not be obtained due to the lack of an X-ray structure of the HadA:FADH^–^:substrate ternary complex (for more see the Discussion section), DFT calculations on the electronic structures and thermodynamic parameters of phenol derivatives could be obtained with reasonable accuracy.^63^–^66^ These parameters were not used alone to pinpoint the HadA reaction mechanism but were employed in conjunction with experimentally measured rate constants to identify factors governing the individual steps of catalysis. As the structures of all phenolic substrates studied are similar in size for all the reactions in Fig. 2 and Table 2, the enzyme active site environment is expected to be the same. Rationales for the use of each parameter for correlation with rate constants are described below.

As the proton dissociation constant (p*K*_a_) indicates the deprotonation ability of compounds, experimental p*K*_a_ values of the phenolic substrates^67^ were used as parameters for correlation with the hydroxylation rate constant (Table 3). If the deprotonation step controls the overall hydroxylation, the hydroxylation rate constant would be higher at lower p*K*_a_ values.

**Table 3:** Thermodynamic and electronic parameters of various phenolic substrates

Substrate	*E*_HOMO_ ^*a*^ (eV)	Energy gap^*a*^ (eV)	Δ*G*_De_^*a*^ (kcal mol^–1^)	Δ*G*_OH_^*a*^ (kcal mol^–1^)	Δ*G*_OH,Overall_^*a*^ (kcal mol^–1^)	p*K*_a_^*b*^	*R* (C_1_–O)^*a*^ (Å)	*R* (O–H)^*a*^ (Å)	C–X BDE^*c*^ (kcal mol^–1^)
Phenol	–0.183	0.106	2.8	–49.1	–46.3	9.99	1.390	0.970	112.9
4-FP	–0.183	0.106	2.5	–42.9	–40.3	9.89	1.389	0.970	127.2
4-CP	–0.186	0.109	1.3	–38.3	–37.0	9.41	1.387	0.970	97.1
4-BP	–0.186	0.109	0.9	–39.0	–38.1	9.37	1.387	0.970	84.0
4-IP	—	—	—	—	—	9.33	—	—	67.0
4-NP	–0.213	0.137	–3.6	–23.3	–27.0	7.15	1.358	0.971	72.5

^*a*^Result from DFT calculation.

^*b*^Experimental p*K*_a_ values from ref. 67.

^*c*^Experimental bond enthalpies of PhX from ref. 65.

On the other hand, to determine the influence of the –OH group transfer on the hydroxylation step, we used the calculated electronic and thermodynamic parameters. In the –OH transfer, phenol derivatives in the phenolate forms were considered nucleophiles and C4a-hydroperoxy-FAD was considered an electrophile. Based on frontier molecular orbital (MO) theory, a lower energy gap between the HOMO energy of phenolate substrates (*E*_HOMO_) and the LUMO energy of C4a-hydroperoxy-FAD (*E*_LUMO_) should facilitate the –OH transfer. The electron distribution of the HOMO of each phenolic substrate and the LUMO of C4a-hydroperoxy-FAD is shown in Fig. S6.† As the enzyme active site is presumably the same in all reactions, the *E*_LUMO_ for C4a-hydroperoxy-FAD is considered as a constant with a calculated value of –0.077 eV. *E*_HOMO_ of the substrates and the energy gap between the *E*_LUMO_ of C4a-hydroperoxy-FAD and the *E*_HOMO_ of the substrates (*E*_LUMO_–*E*_HOMO_) are summarized in Table 3. If the hydroxylation reaction is controlled by the –OH transfer, the hydroxylation rate constant should be higher when the HOMO–LUMO energy gap (*E*_LUMO_–*E*_HOMO_) is lower.

**Scheme 5:**
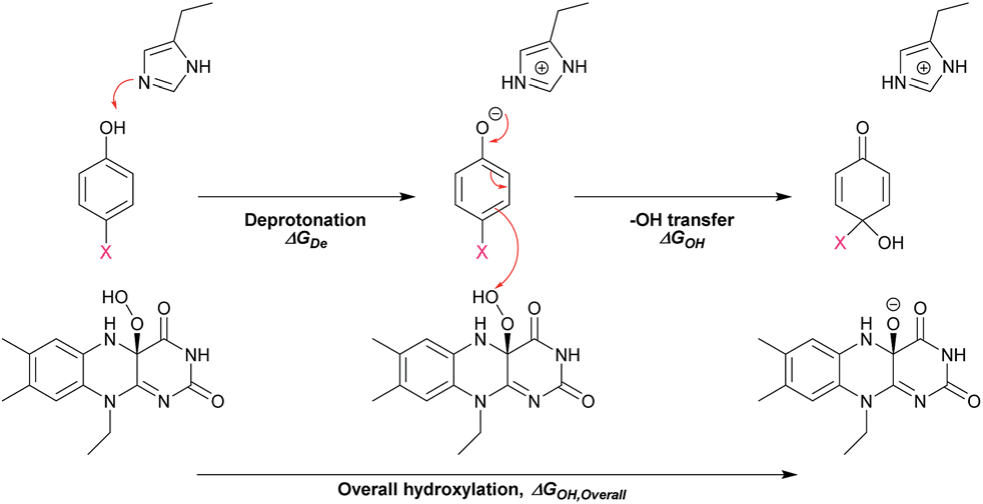
The proposed model for calculation of Gibbs free energies of hydroxylation (Δ*G*_OH_).

In addition, DFT calculations were performed to obtain the Gibbs free energy of hydroxylation (Δ*G*_OH_) to determine the thermodynamic driving force for the reaction. Modelling details of the calculation are provided in Fig. S7.† Based on the apoenzyme structures of TftD and TcpA (HadA homologs) and ligand docking of a phenolic substrate and FAD using the ternary complex structure of a hydroxylase HpaB from *Thermus thermophilus* HB8 as a guideline, an active site His was proposed to be a key catalytic base for initiation of proton abstraction^27^,^68^,^69^ (Scheme 5). The Gibbs free energies of separately calculated phenolic substrates, C4a-hydroperoxy-FAD and a proposed His catalytic residue were combined to obtain the Gibbs free energies of deprotonation (Δ*G*_De_), –OH transfer (Δ*G*_OH_), and overall hydroxylation reaction (Δ*G*_OH,Overall_) based on the reaction in Scheme 5. The results are summarized in Table 3. As the thermodynamic driving force is one of the factors that can facilitate a faster reaction,^70^ if the –OH transfer controls the overall hydroxylation, the rate constants of hydroxylation may be higher with lower Δ*G*_OH_. As we utilized only the B3LYP/6-31G(d) basis set to calculate Δ*G*, the values obtained in this report may not be highly accurate. It requires a more precise method such as those previously reported^63^–^66^,^71^–^73^ for more accurate calculations. However, because all substrates were calculated using the same basis set, the values obtained can represent the trend of Δ*G* changes which are good enough to evaluate whether the individual step is dependent on particular Δ*G* values.

Although the group elimination step in the reaction of HadA with phenol containing *para*-substituents is likely to occur *via* heterolytic C–X bond cleavage (Scheme 4), the bond dissociation energy (BDE) for the corresponding homolytic cleavage can be used to represent the strength of the C–X bond. The lower value of BDE indicates a weaker C–X bond and greater stability of X^–^. Thus, the experimental bond dissociation energy (BDE)^65^ was used for correlation with the rate constants of the group elimination step (Table 3). If BDE is a major factor controlling the group elimination step, the rate constants of group elimination would be greater when the BDE values are smaller.

#### Substrate deprotonation controls the hydroxylation step

**Fig. 3:**
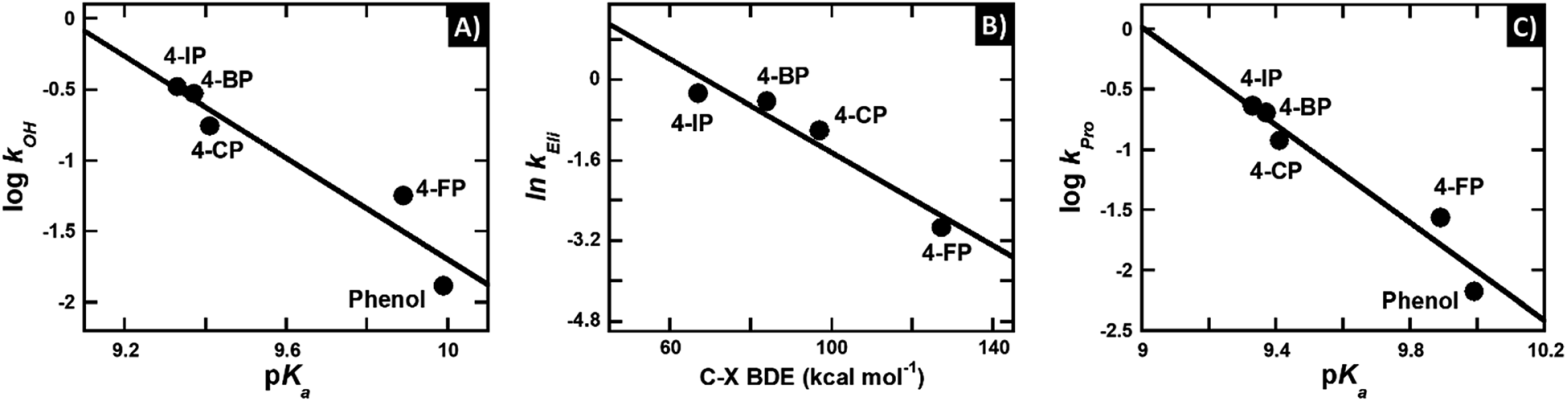
Quantitative structure–activity relationship of the reaction of HadA with various *para*-substituted phenols. (A) A plot of the logarithms of rate constants of hydroxylation (*k*_OH_) *versus* p*K*_a_ of para-substituted phenols yields a slope (*β*-value) of –1.8 and *R* = 0.96. (B) A plot of the natural logarithms of group elimination rate constants (*k*_Eli_) *versus* bond dissociation energy (BDE) of C–X shows a linear relationship with a slope of –0.05 and *R* = 0.96. (C) A plot of the logarithms of product formation rate constants (*k*_Pro_) *versus* p*K*_a_ of *para*-substituted phenols yields a linear relationship with a slope of –2.0 and *R* = 0.97.

The rate constants of hydroxylation were correlated with the thermodynamic and electronic parameters mentioned above. A plot of the logarithms of the hydroxylation rate constants (*k*_OH_) *versus* the phenolic substrate p*K*_a_ values obtained using the Brønsted equation (eqn (5)) shows a linear relationship with higher rate constants at lower p*K*_a_ values, consistent with a slope (*β*-value) of –1.8 and an *R* value of 0.96 (Fig. 3A).

5 log *k* = *β*·p*K*_a_ + *C*.

The negative *β*-value suggests that there is a significant negative charge characteristic developed during the formation of the transition state (*i.e.* the step with the highest activation energy) which controls the overall rate of hydroxylation. The hydroxylation rate constant is higher at lower p*K*_a_'s, and this indicates that the highest activation energy is lower when the p*K*_a_ value of the phenolic substrate is lower. These data agree well with the calculated Gibbs free energy of deprotonation (Δ*G*_De_) in Table 3 in that *k*_OH_ is higher when the deprotonation step has a lower Δ*G*_De_, or is more exergonic (Fig. S8A†). Because the lower p*K*_a_ value directly indicates easier phenolic deprotonation, these data suggest that deprotonation of the phenolic substrate is the step that controls the overall process of hydroxylation.

In contrast, the correlation of logarithms of the hydroxylation rate constants (*k*_OH_) *versus* Δ*G*_OH_, Δ*G*_OH,Overall_, or the energy gap of *E*_LUMO_–*E*_HOMO_ does not show an increase in the *k*_OH_ when the energy associated with the –OH transfer is lower (Fig. S8B–D†). These results suggest that the hydroxylation step is not controlled by the –OH transfer process. This analysis agrees well with the analysis of the hydroxylation rate constant *versus* the p*K*_a_ value above in that the HadA hydroxylation is controlled by the deprotonation process of phenolic substrates. The findings for the HadA reaction are different from the mechanism of hydroxylation previously reported for flavin-dependent monooxygenases (hydroxylases) which catalyze only the hydroxylation of aromatic compounds in that for general flavin-dependent hydroxylases, their hydroxylation mechanism was proposed to be controlled by the –OH transfer step.^60^–^62^,^74^–^77^

**Chart 2:**
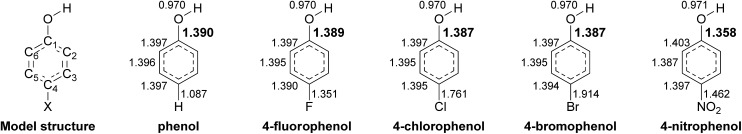
Bond lengths of HP and NP substrates obtained from structural optimization using B3LYP/6-31G(d).

In order to further address the physical parameters which promote deprotonation of the phenolic substrates, we explored the bond length of the C_1_–O bond of all the substrates from DFT calculations. The calculation results indicate that phenols with substituents (especially 4-NP) have shorter C_1_–O bonds than phenol without any substituents (Chart 2 and Table 3). The plots of the logarithm of the hydroxylation rate constants (*k*_OH_) *versus* C_1_–O lengths also correlated well with an *R* value of 0.96 (Fig. S8E†). These data indicate that the reaction occurs faster when the substrate C_1_–O bond is shorter. The shorter C_1_–O bond supports better electron delocalization into the aromatic ring, translating to easier deprotonation and subsequently, lower p*K*_a_ values. As the transition state has a negative charge characteristic (the Brønsted plot in Fig. 3A), the greater ability of the compounds to delocalize electrons into the aromatic ring would directly lower the activation energy gap. Therefore, the hydroxylation step is faster with substrates that have lower p*K*_a_ values because they have the ability to delocalize and thus stabilize the transition state of the reaction.

#### Group elimination is controlled by bond dissociation energy

A plot of the natural logarithms of halogen elimination rate constants (*k*_Eli_) for the reaction of HadA with 4-FP, 4-CP, 4-BP, and 4-IP *versus* BDE yielded a linear relationship in which lower BDE values resulted in higher rate constants of group elimination with *R* = 0.96 (Fig. 3B). The analysis indicates that the group elimination in the reaction of HadA is directly dependent on the strength of the C–X bond or the stability of the leaving group.

#### The hydroxylation has a higher activation energy than the group elimination step and the overall product formation is controlled by substrate deprotonation

As the overall product formation of *para*-substituted phenols consists of hydroxylation and group elimination steps, we identified which step has a higher activation energy or controls the overall process of product formation using rate constant values and QSAR. The results in Table 2 indicate that the rate constants of hydroxylation are lower than the rate constants of group elimination for most substrates (4-CP, 4-BP, and 4-IP), indicating that the activation energy of hydroxylation is larger than that of the group elimination and the overall product formation is controlled by the hydroxylation step. The QSAR analysis also indicates that a plot of the logarithms of the rate constants obtained from rapid quench techniques (*k*_Pro_) *versus* p*K*_a_ values yielded a good correlation (*R* = 0.97) (Fig. 3C), similar to the QSAR analysis of the hydroxylation (Fig. 3A). This analysis suggests that the overall product formation is controlled by the hydroxylation step, in agreement with the approach comparing the rate constants mentioned above. Altogether, the results suggest that the hydroxylation step has a higher activation energy than the group elimination step and that the overall process is controlled by substrate deprotonation.

### Mechanistic implications on the HadA reaction

**Fig. 4:**
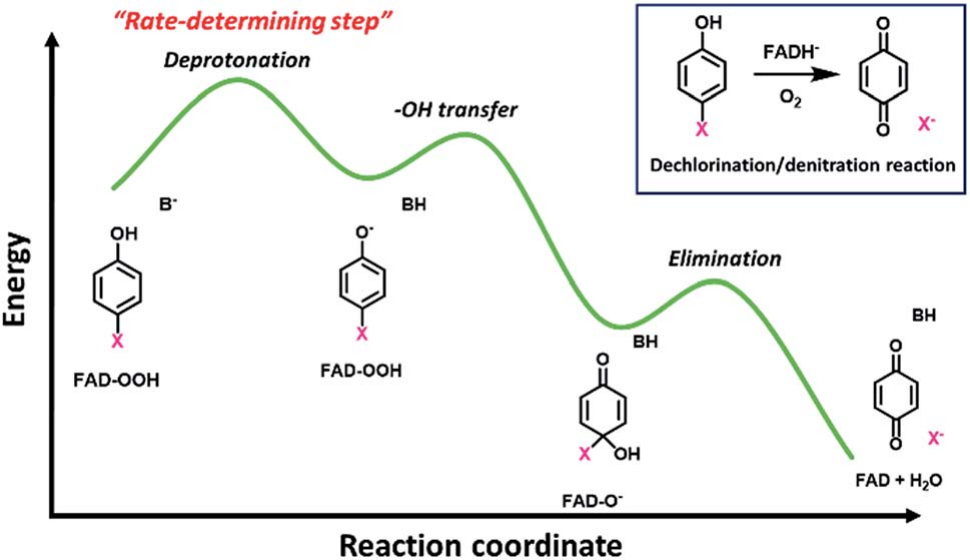
Proposed energy diagram for oxidative dehalogenation and denitration of HadA based on QSAR analysis.

The overall process of oxidative dehalogenation and denitration consists of hydroxylation and group elimination steps. The hydroxylation process is controlled by substrate deprotonation, not –OH transfer, indicating that the substrate deprotonation has a higher energy barrier than the –OH transfer. For the group elimination, the process is controlled by the ability of the C–X bond to break. Based on the analysis of rate constants associated with the individual steps and QSAR, the substrate deprotonation step should be the step with the highest energy barrier for the overall process of oxidative dehalogenation and denitration by HadA. The overall energy diagram of HadA reaction is proposed in Fig. 4. Our results herein have shown for the first time the mechanistic factor governing the individual steps of oxidative dehalogenation or denitration by a flavin-dependent monooxygenase.

## Discussion

Our findings indicate that HadA is a flavin-dependent monooxygenase that exhibits dual oxidative dehalogenation and denitration activities. Investigation of HadA reactions with phenol, 4-FP, 4-CP, 4-BP, 4-IP, 4-NP, 2-CP, 2-IP, and 2-NP using combined transient kinetic approaches of stopped-flow absorbance and fluorescence and rapid-quench flow techniques can unequivocally identify the rate constants associated with the individual steps of the HadA reaction. These kinetic data in correlation with the thermodynamic and electronic parameters of the substrates obtained from DFT calculations together with the experimental physico-chemical parameters were used in QSAR analysis to identify the key factors governing each reaction step of the HadA reaction. Analysis has shown that unlike the mechanism of other hydroxylases which catalyze phenol hydroxylation alone,^60^–^62^,^74^–^77^ the hydroxylation of HadA is controlled by substrate deprotonation, not –OH group transfer. For the group elimination, the process is controlled by the C–X bond dissociation energy. The QSAR analysis has shown that the overall oxidative dehalogenation and denitration of HadA is controlled by the hydroxylation step, particularly the substrate deprotonation. This report is the first experimental investigation of the mechanistic factor governing oxidative dehalogenation and denitration in a flavin-dependent monooxygenase.

The dual dehalogenation and denitration activities of HadA towards HPs and NPs to generate benzoquinone or hydroquinone derivatives suggest that the enzyme should be useful as a biocatalyst in the detoxification of HPs and NPs because only a single enzyme can be used to convert two different classes of toxic compounds into common metabolites (hydroquinones or benzoquinones) which can be assimilated by many common soil microbes. Previously, a single-component flavoenzyme, PcpB from *S. chlorophenolicum*, was also reported to be able to catalyze the elimination of a halogen, nitro, or cyano group from phenolic compounds.^35^ Some of the two-component enzymes from the degradation pathways of NPs such as NpdA2 from *Arthrobacter* sp. strain JS443 (42.4% identity to HadA)^45^ and NpsA1 from *Rhodococcus* sp. strain PN1 (42.5% identity to HadA)^43^ were also reported to utilize some CPs. However, most of these studies were carried out with crude lysate or partially purified enzymes. Our work reported herein has clearly established *in vitro* activities of HadA toward HPs and NPs and demonstrated that the enzyme can be used to detoxify HPs and NPs even in a mixture of compounds (Fig. 1).

The rate constants associated with the individual steps derived from three transient kinetic techniques (stopped-flow spectrophotometry, fluorescence, and rapid-quench flow) were very useful for identifying the key factors governing the overall process of oxidative dehalogenation/denitration. Our work provides the first report in which the reaction mechanism of enzymatic oxidative dehalogenation was investigated experimentally. Some of the results obtained contradict computational calculations previously reported for the dechlorination reaction of TftD, a homolog of HadA. In a previous study of TftD, the ternary complex used for the QM/MM calculation was the model of flavin and substrates docked into the apo-structure of TftD.^59^ Results from the calculation showed that the hydroxyl group transfer from C4a-hydroperoxy-FAD to 2,4,5-TCP and 2,5-DCHQ has the highest energy barrier among all process steps (16.9 and 19.7 kcal mol^–1^), while the barrier of the chloride elimination step is very small (0.3 and 0.2 kcal mol^–1^). The calculation did not detect a high energy barrier of substrate deprotonation and explained the deprotonation and –OH transfer as a concerted process.^59^ These results are different from our results in two aspects. First, as the rate constant of Cl^–^ elimination is only twice as high as the corresponding value of the hydroxylation step (Table 2), although our data agree with the TftD calculations in that the Cl^–^ elimination has a lower energy barrier than the hydroxylation step, it does not support such a large difference in activation energy of these two steps as in the computational work proposed. Second, our QSAR identified substrate deprotonation (not –OH transfer) as the step that governs the dehalogenation; the rate constants of product formation or hydroxylation can be directly increased when the p*K*_a_ value of the substrate is lower. Calculations of the TftD reaction did not identify substrate deprotonation as a discrete step that has a high energy barrier.^59^ A potential energy surface scan along both reaction coordinates of the proton transfer and the –OH transfer would be necessary to address this point.^59^ Moreover, the system used in their calculations was obtained from modelling flavin and the substrate into the apoenzyme structure. As the active site structure of TftD is quite spacious, it is difficult to perform an accurate ligand docking process because many orientations of flavin and substrate binding are possible. These might have caused the discrepancy between the QM/MM results in the previously published work and our experimental work. In our opinion, the X-ray structure of the enzyme:FADH^–^:substrate ternary complex is required for accurate analysis of QM or QM/MM calculations of HadA or TftD reactions.

The finding that the hydroxylation step in oxidative dehalogenation/denitration in HadA is controlled by substrate deprotonation is different from reactions of other flavin-dependent monooxygenases that catalyze mainly hydroxylation.^60^–^62^,^74^–^77^ Among all the reaction steps of HadA, substrate deprotonation is likely the step with the highest energy barrier because the factor which facilitates substrate deprotonation (*i.e.* lowering the phenolic group p*K*_a_) directly correlated with an increase in the hydroxylation and product formation rate constants (Fig. 3A and C). Although deprotonation has been considered to be important for activating the substrate for electrophilic aromatic substitution in flavin monooxygenases,^60^,^78^ thus far, there is no clear evidence to suggest that the deprotonation should be the crucial step like in this work. Analysis of optimized electronic structures of substrates indicates that substrates with lower p*K*_a_ values have shorter C_1_–O bonds (Fig. 4). This effect allows electron delocalization from the O^–^ of the phenolate moiety into the C_4_ position of the aromatic ring to facilitate the electrophilic aromatic substitution reaction between phenolate and the C4a-hydroperoxy-FAD intermediate (Scheme 4).

The QSAR results for the HadA reaction are clearly different from those of flavin-dependent monooxygenases that catalyze mainly the hydroxylation of aromatic compounds *via* the electrophilic aromatic substitution mechanism. Previous investigations of *p*-hydroxybenzoate hydroxylase (PHBH) from *Pseudomonas fluorescens* suggest that the –OH transfer from the C4a-hydroperoxy-FAD intermediate to aromatic substrates is the key step that controls the hydroxylation.^78^ The *k*_cat_ values for the reaction of PHBH with a series of fluorine substituted *p*-hydroxybenzoate substrates were measured and the values were used in QSAR analysis for correlation with the *E*_LUMO_–*E*_HOMO_ energy gap. Unlike the results of the HadA reaction, the *k*_cat_ of PHBH is higher with a lower molecular orbital (MO) energy gap, suggesting that the –OH transfer is the rate-determining step for PHBH.^72^–^75^ For the reaction of phenol hydroxylase, the *k*_cat_ values of the enzyme with a series of fluorine-substituents are also higher when the MO energy gap of C4a-hydroperoxy-FAD and substrates is lower, suggesting that the –OH transfer is also the major step controlling the reaction of phenol hydroxylase.^73^ The correlation of the phenol hydroxylase reaction with a set of halogenated phenols with the *E*_LUMO_–*E*_HOMO_ energy gap also indicates that the *k*_cat_ value is increased with a lower energy gap.^61^ A direct correlation between the hydroxylation rate constant and the *E*_LUMO_–*E*_HOMO_ energy gap was also found in the reaction of 2-methyl-3-hydroxypyridine-5-carboxylic acid oxygenase (MHPCO), a single-component FAD-dependent enzyme that catalyzes hydroxylation plus a ring-cleavage reaction of 2-methyl-3-hydroxypyridine-5-carboxylic acid (MHPC) and also 5-hydroxynicotinic acid (5-HN). The higher hydroxylation rate constant was obtained with a lower energy gap between the HOMO of the nucleophile (5-HN) and the LUMO of the electrophile (8-substituted-FAD analogues).^74^ For the reaction of pentachlorophenol hydroxylase (PcpB) from *S. Chlorophenolicum*, the rate of substrate consumption is higher with higher substrate binding affinity.^36^

The substrate deprotonation mechanism in HadA and two-component monooxygenases is different from single-component monooxygenases. The substrate deprotonation mechanism of PHBH is mediated through a continuous H-bond network from His72 on the enzyme surface through the active site Tyr201.^79^,^80^ The deprotonation of *p*-hydroxybenzoate (PHB) is important for the –OH group transfer from C4a-hydroperoxy-FAD to the substrate *via* an electrophilic aromatic substitution mechanism.^81^ Computational calculations suggest that the active site environment facilitates the elongation of the O–O bond of C4a-hydroperoxyflavin to enhance the –OH transfer reaction.^62^ QM/MM studies of phenol hydroxylase suggest that the phenol substrate binds to Asp54 in the enzyme active site facilitating substrate deprotonation to the phenolate form.^61^ For MHPCO, the hydroxypyridine structure of MHPC or 5HN binds to the enzyme in the deprotonated form.^82^,^83^ The enzyme active site environment, particularly Tyr223 and Tyr82, is important for ensuring substrate deprotonation.^84^

A HadA homology model was built based on the apoenzyme structure of TcpA (pdb: 4G5E)^69^ and TftD (pdb: ; 3HWC)^27^ (Fig. S7†). The HadA model was aligned with the ternary complex structure of 4-hydroxyphenylacetate 3-monooxygenase or HpaB from *Thermus thermophilus* HB8 (pdb: ; 2YYJ)^68^ bound with FAD and 4-hydroxyphenylacetate to identify the HadA active site area. The alignment identified His290 as a potential active site residue which may potentially serve as a catalytic base to abstract a proton from the substrate –OH. In another two-component flavin-dependent monooxygenase, *p*-hydroxyphenylacetate hydroxylase (C_2_), His 120 was identified as a residue which potentially serves as an active site base to abstract a proton from the phenolic moiety.^85^,^86^ Overall, the key catalytic features to deprotonate the phenolic substrate to facilitate the hydroxylation reaction in two-component and single-component monooxygenases are completely different.

The QSAR analysis of rate constants associated with group elimination (*k*_Eli_) in the HadA reaction indicates that this step is directly dependent on the strength of the C–X bond which can be represented by the bond dissociation energy (BDE) of C–X. This QSAR analysis also supports our model in which the group elimination is depicted as another discrete step apart from the hydroxylation and disagrees with the results of the QM/MM calculations of TftD reaction in which the chloride elimination step is almost barrier-less.^59^ Interestingly, the QSAR trend in the HadA system is similar to the reactivity of a series of halogenated substrates catalyzed by a reductive dehalogenase, human iodotyrosine deiodinase (hIYD). hIYD can catalyze dehalogenation of I-Tyr, Br-Tyr, and Cl-Tyr, but not F-Tyr.^22^ The overall rates of dehalogenation in the oxidative half reaction (*k*_ox_) of hIYD were measured with *k*_ox_ of Cl-Tyr being ∼20 fold lower than that of I-Tyr.^22^,^23^ In hIYD, the reaction does not give a signal which allows measurement of individual rate constants associated with the group elimination step.

Overall, our findings in this report indicate that the substrate deprotonation controls overall enzymatic dehalogenation and denitration processes. Future work on enzyme engineering should focus on HadA evolution to increase the ability of the active site environment to promote substrate deprotonation in order to create HadA variants with higher efficiency in catalyzing dehalogenation and denitration of toxic compounds than the wild-type enzyme.

## Conclusions

The HadA monooxygenase from *R. pickettii* DTP0602 performs dual functions of oxidative dechlorination and denitration and can be used to detoxify a broad range of halogenated phenols (HPs) and nitrophenols (NPs). Using a combination of transient kinetic techniques, density functional theory and quantitative structure–activity relationship (QSAR), the substrate deprotonation step was identified as the step that controls the overall reaction of HadA and probably has the highest energy barrier which is unique among flavin-dependent hydroxylases.

## Conflicts of interest

There are no conflicts to declare.

## Supplementary Material

Supplementary information
