# Supplementary material for: Oxidative dehalogenation and denitration by a flavin-dependent monooxygenase is controlled by substrate deprotonation
Source: Chem Sci. 2018 Aug 8;9(38):7468–82. doi: 10.1039/c8sc01482e (PMC6180312; doi:10.1039/c8sc01482e)
Supplement: Supplementary file 1 [file SC-009-C8SC01482E-s001.pdf]

Electronic Supplementary Material (ESI) for Chemical Science.
This journal is © The Royal Society of Chemistry 2018

Supporting Information

Oxidative Dehalogenation and Denitration by a Flavin-dependent Monooxygenase is Controlled by Substrate Deprotonation

Panu Pimviriyakul[a,b], Panida Surawatanawong[c], and Pimchai Chaiyen[a*]

[a]School of Biomolecular Science and Engineering, Vidyasirimedhi Institute of Science and Technology (VISTEC), Wangchan Valley, Rayong, 21210, Thailand. [b]Department of Biochemistry and Center for Excellence in Protein and Enzyme Technology, Faculty of Science, Mahidol University, Bangkok, 10400, Thailand. [c]Department of Chemistry and Center of Excellence for Innovation in Chemistry, Faculty of Science, Mahidol University, Rama 6 Road, Bangkok, 10400 Thailand.

*Corresponding author:
Pimchai Chaiyen, Department of Biomolecular Science and Engineering, School of Biomolecular Science and Engineering, Vidyasirimedhi Institute of Science and Technology (VISTEC), Rayong, 21210, Thailand.

*E-mail: pimchai.chaiyen@vistec.ac.th. Phone: +66-896695410

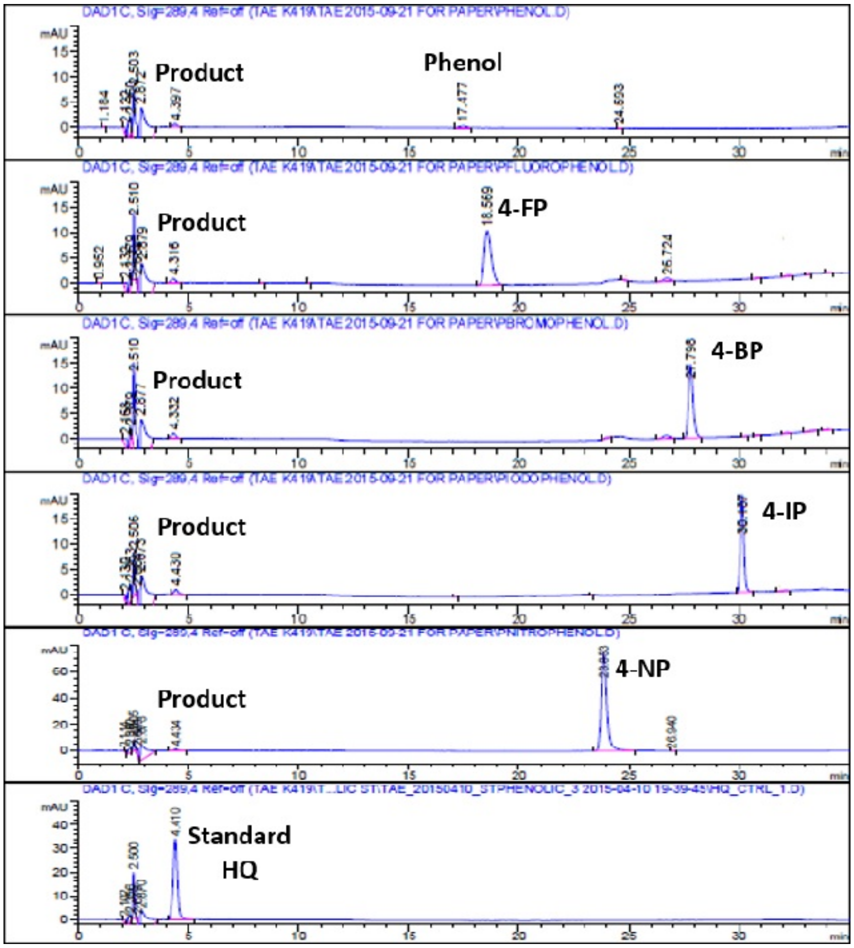

Figure S1. HPLC chromatograms of single turnover reactions of HadA with phenol and *para*-substituted phenols. HPLC/DAD analysis was performed using a Nova-Pak® C18 reverse-phase column with a 4 µm particle size and 3.9 x 150 mm column size. The mobile phase was a gradient of water/methanol containing 0.1% formic acid with a flow rate of 0.5 ml/min. Mobile phase gradients for analyses were carried out from 10-30% methanol for phenol, 10-70% methanol for 4-FP, 4-BP, and 4-IP, and 10-50% methanol for 4-NP. Chromatograms of phenol, 4-FP, 4-BP, 4-IP, 4-NP, and HQ were monitored at retention times of 17.5, 18.6, 27.8, 30.2, 23.8, and 4.4 min, respectively. Products from all reactions have the same retention time as that of the standard HQ.

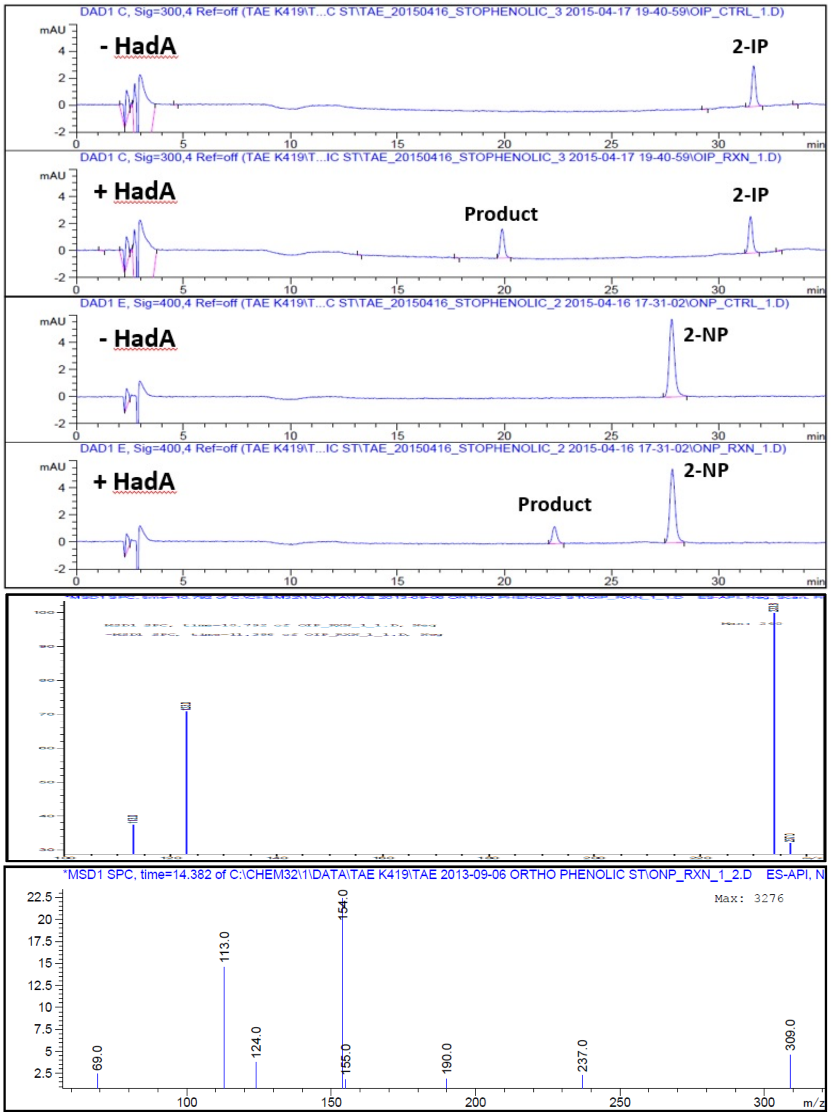

Figure S2. HPLC chromatograms and mass spectra of products from single turnover reactions of HadA with *ortho*-substituted phenols. Mobile phase was a gradient of H_2O/methanol containing 0.1 % formic acid with a flow rate of 0.5 ml/min. The gradient was carried out from 5-50 % methanol. (*A)* Hydroxylated products of 2-IP and 2-NP were monitored at 300 and 400 nm. Mass spectra of the products from 2-IP *(B)* and 2-NP *(C)* reactions showed molecular masses that were correlated with iodohydroquinone (IHQ, *m/z* of 236.0) and nitrohydroquinone (NHQ, *m/z* of 154.0), respectively.

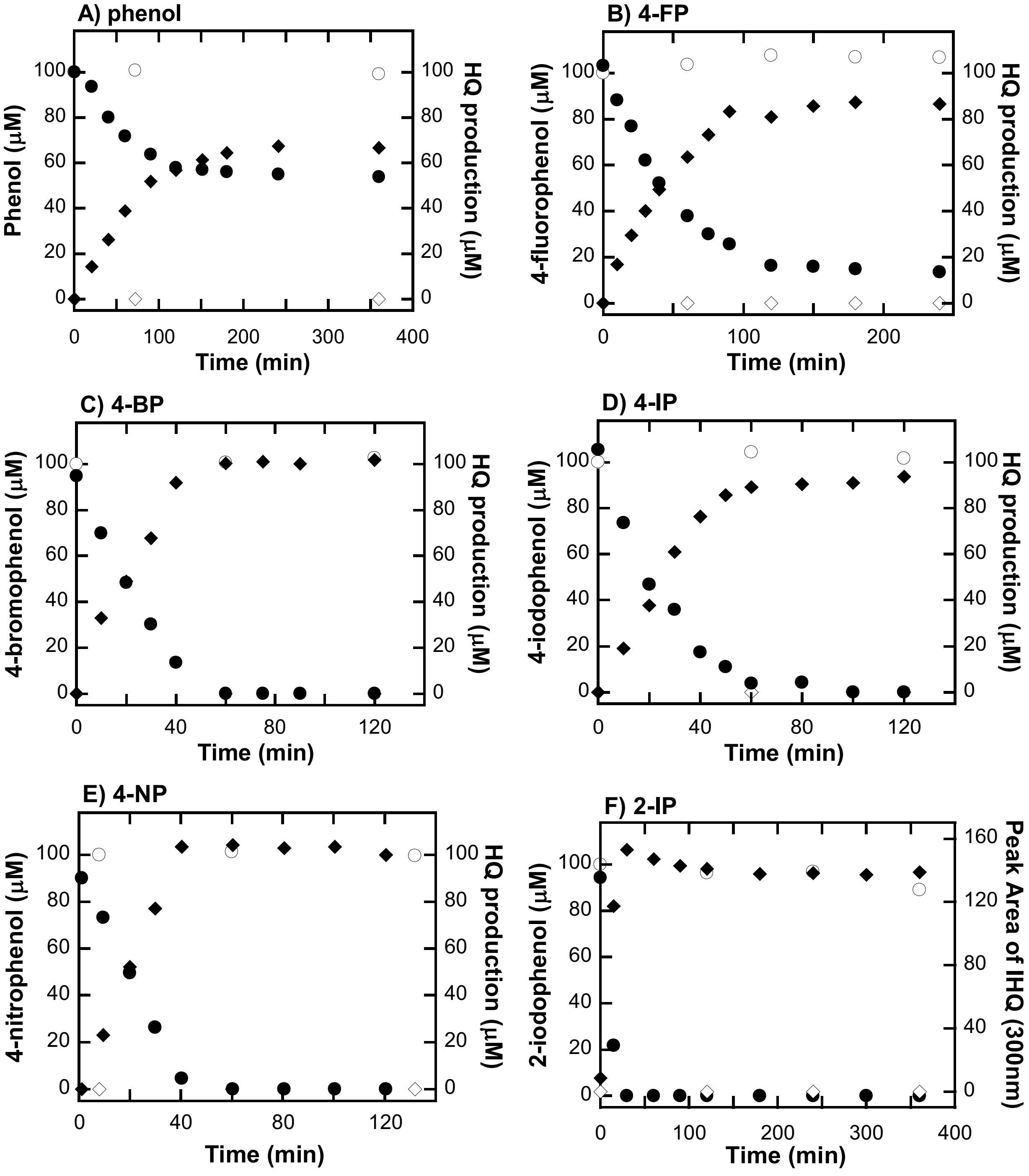

A) phenol
Phenol (μM)
HQ production (μM)
Time (min)
B) 4-FP
4-fluorophenol (μM)
HQ production (μM)
Time (min)
C) 4-BP
4-bromophenol (μM)
HQ production (μM)
Time (min)
D) 4-IP
4-iodophenol (μM)
HQ production (μM)
Time (min)
E) 4-NP
4-nitrophenol (μM)
HQ production (μM)
Time (min)
F) 2-IP
2-iodophenol (μM)
Peak Area of IHQ (300nm)
Time (min)

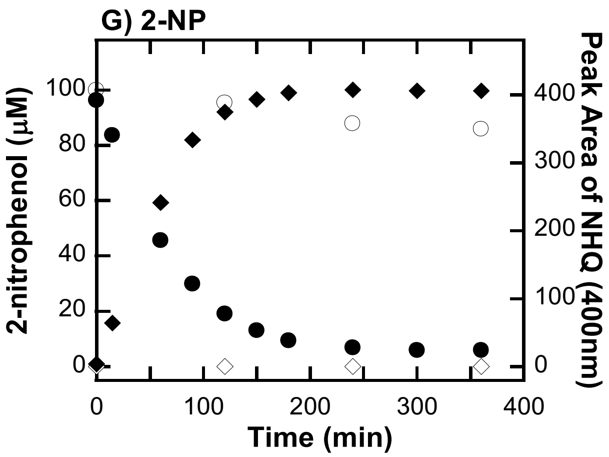

Figure S3. Bioconversion of phenolic substrates by multiple turnover reactions of HadA. Plots of the amount of substrate remaining and products generated from reactions of *(A)* phenol, *(B)* 4-FP, *(C)* 4-BP, *(D)* 4-IP, *(E)* 4-NP, *(F)* 2-IP, and *(G)* 2-NP are shown over a time course of 2-10 hr. Empty circles indicate substrate remaining in the control reaction without HadA. Filled circles indicate substrate remaining during multiple turnover reactions. Empty diamonds indicate product amount in the control reaction without HadA. Filled diamonds indicate product formed during multiple turnover reactions.

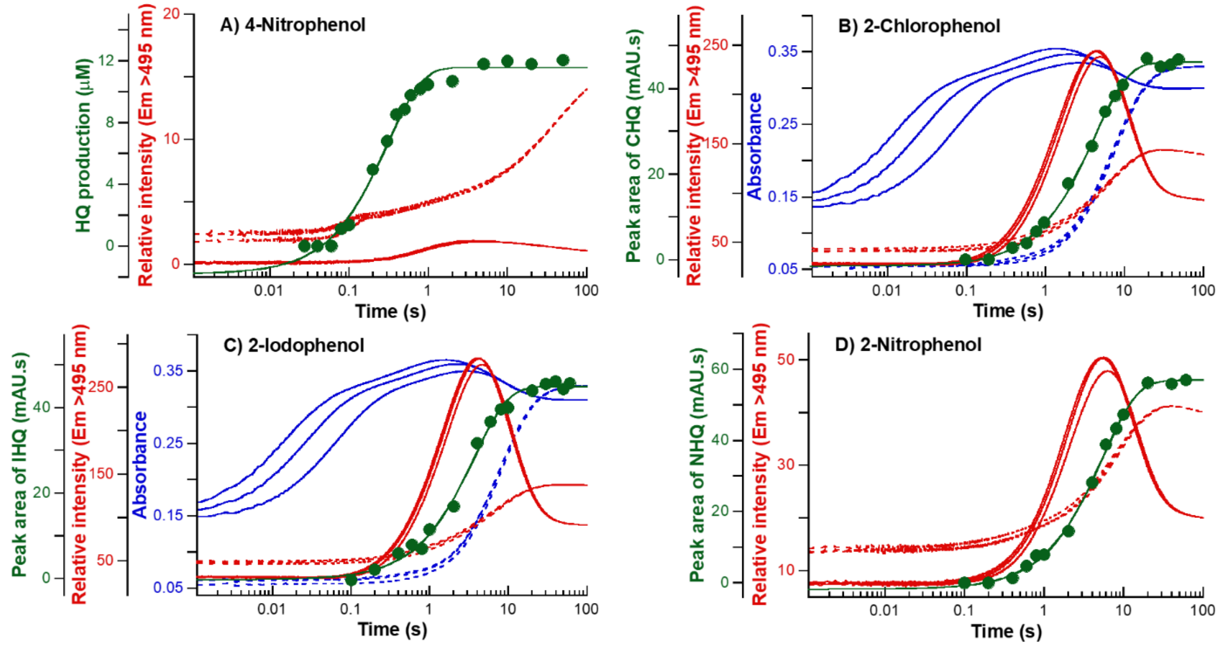

Figure S4. Kinetic traces of the reactions of HadA:FADH⁻ with 4-NP, 2-CP, 2-IP, and 2-NP. Kinetic traces obtained from single turnover reactions of a HadA:FADH⁻ binary complex (75 µM and 25 µM, respectively) with *(A)* 4-NP, *(B)* 2-CP, *(C)* 2-IP, and *(D)* 2-NP (0.8 mM each) and 0.13, 0.31, and 0.61 mM oxygen in 20 mM HEPES pH 7.5 at 25°C. *Blue lines* are kinetic traces detected by stopped-flow spectrophotometry at wavelengths 380 (solid line) and 450 nm (dashed line). *Red lines* are fluorescence signal detected by a stopped-flow apparatus using the excitation wavelength of 380 nm (solid line) and 450 nm (dashed line) with emission wavelengths >495 nm. The *green line* is a calculated exponential curve that is correlated with kinetics of product formation monitored by rapid-quench flow (RQF) and HPLC/DAD techniques.

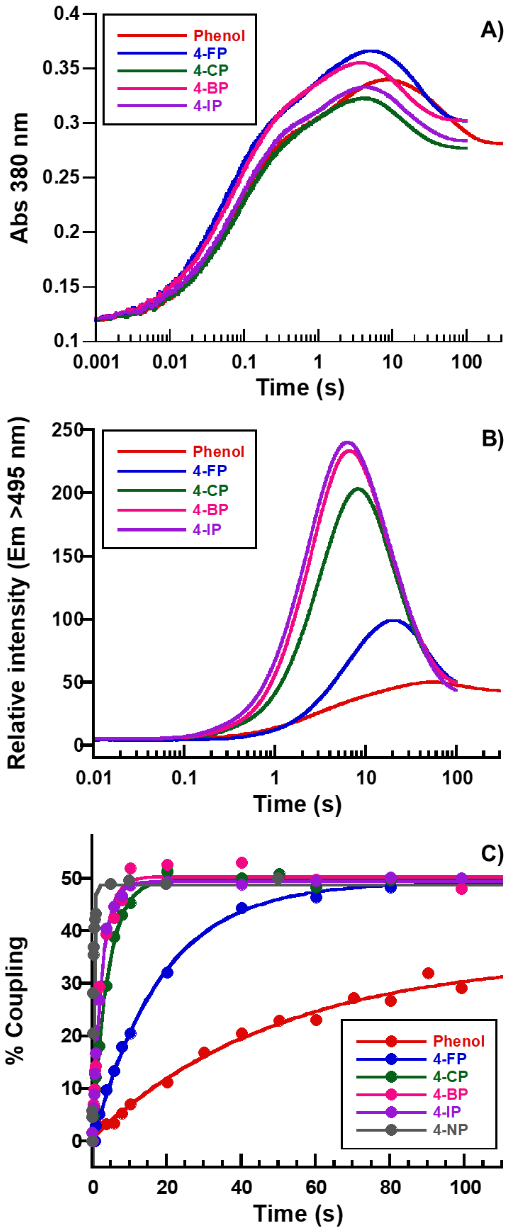

Figure S5. Comparison of kinetics of the reaction of HadA:FADH⁻ with various substrates. (A), (B), and (C) are kinetic traces obtained from the HadA:FADH⁻ binary complex reacting with aerobic phenol (red lines), 4-FP (blue lines), 4-CP (green lines), 4-BP (pink lines), 4-IP (purple lines), and 4-NP (grey lines) detected by stopped-flow spectrophotometry at 380 nm, stopped-flow fluorescence excited at 380 nm, and RQF, respectively.

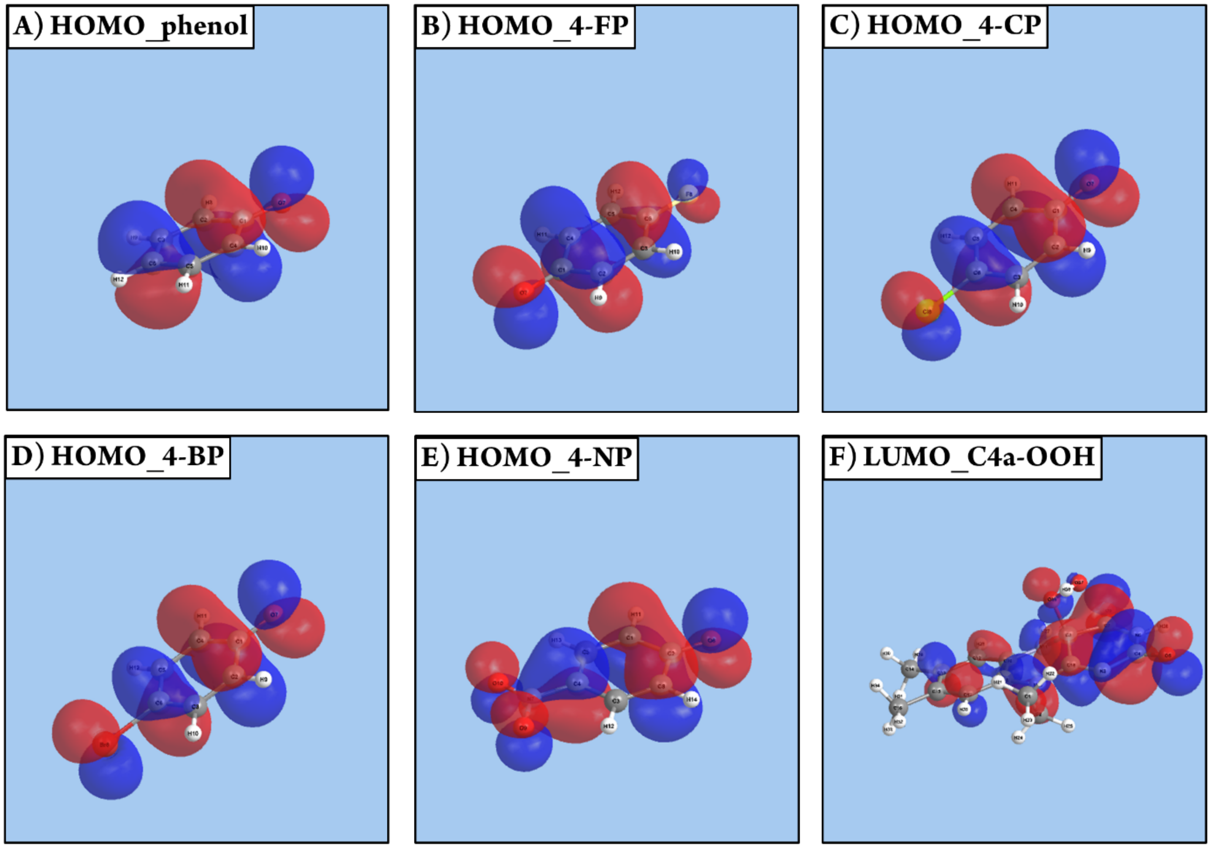

Figure S6. Calculated HOMO and LUMO plots. HOMO plots of *(A)* phenol, *(B)* 4-FP, *(C)* 4-CP, *(D)* 4-BP, and *(E)* 4-NP. LUMO plot of *(F)* C4a-hydroperoxy-FAD intermediate.

Modeling of HadA structure with C4a-hydroperoxy-FAD and substrate bound

The HadA apoenzyme structure was modelled based on X-ray structures of two homologues, TcpA from *R. eutropha* JMP134 (86.7 % sequence identity to HadA) and TftD from *B. cepacia* AC1100 (64.2% sequence identity to HadA)[A-B]. As none of these enzymes has FAD nor substrate bound to the active site, the FAD and phenolic substrate-bound structure of 4-hydroxyphenylacetate 3-monooxygenase or HpaB from *Thermus thermophilus* HB8 (PDB: 2YYJ)[C] (a two-component flavin-dependent monooxygenase that catalyzes hydroxylation of 4-hydroxyphenyl benzoate) was used as a guide to optimize ligand binding of C4a-hydroperoxy-FAD and substrates. The alignment has identified His290 of HadA as a potential base residue that interacts closely with the substrates (Figure S7).

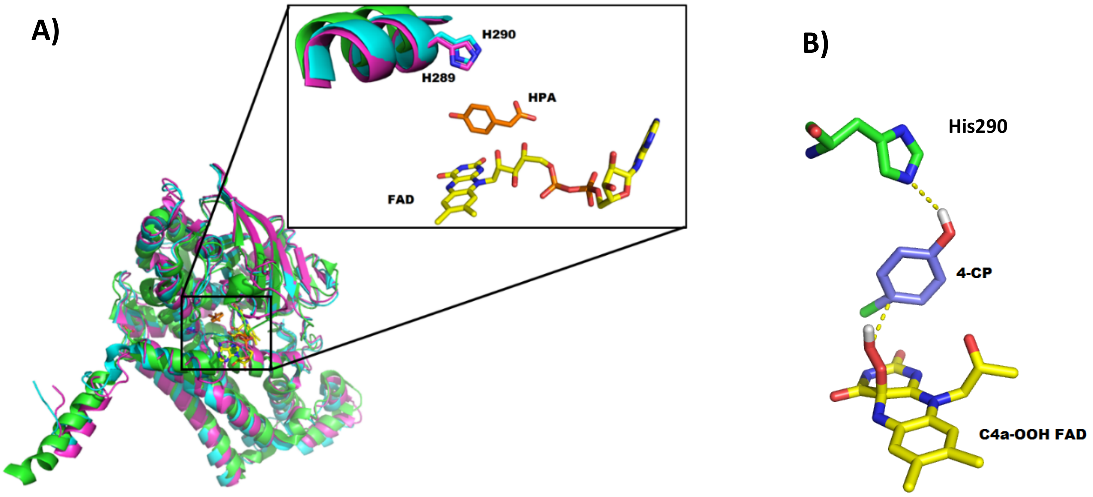

Figure S7. Modeling of the HadA structure with C4a-hydroperoxy-FAD and substrate bound. *(A)* Structure alignment of apo structures of TcpA (PDB: 3HWC, magenta) and TftD (PDB: 4G5E, cyan) with HpaB (PDB: 2YYJ, green) yielding rms of 1.70 and 1.90, respectively. His290 of TcpA and His289 of TftD are closed to HPA and FAD of HpaB. *(B)* Modelled structures of C4a-hydroperoxy-FAD and 4-CP of HadA constructed based on alignment suggest that His290 is a potential catalytic base for deprotonation of the phenol substrate.

REFERENCES

A) R. P. Hayes, B. N. Webb, A. K. Subramanian, M. Nissen, A. Popchock, L. Xun and C. Kang, *Int. J. Mol. Sci.* 2012, **13** (8), 9769-9784.

B) B. N. Webb, J. W. Ballinger, E. Kim, S. M. Belchik, K. S. Lam, B. Youn, M. S. Nissen, L. Xun and C. Kang, *J. Biol. Chem.* 2010, **285** (3), 2014-2027.

C) S. H. Kim, T. Hisano, K. Takeda, W. Iwasaki, A. Ebihara and K. Miki, *J. Biol Chem.* 2007, **282** (45), 33107-33117.

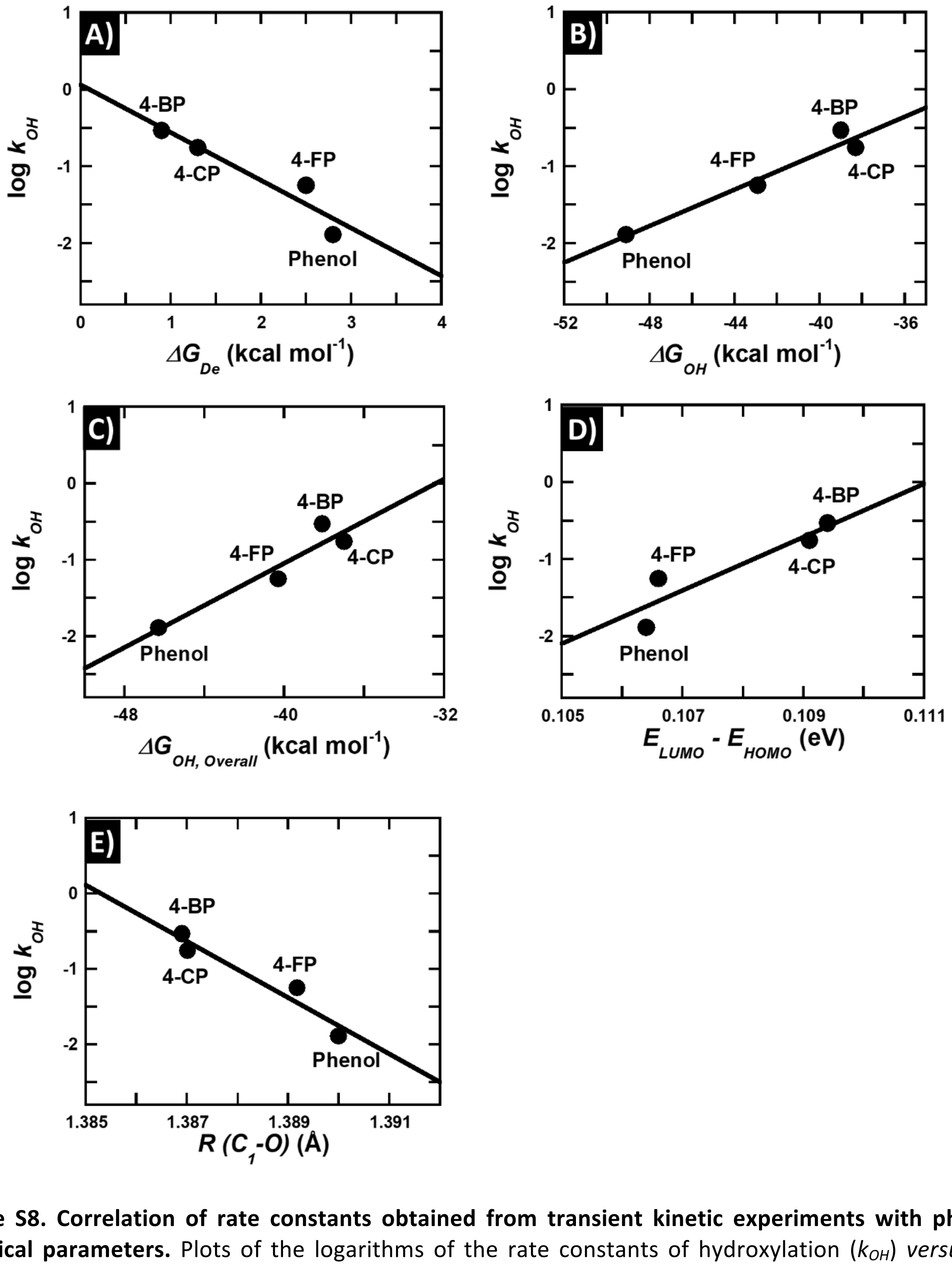

Figure S8. Correlation of rate constants obtained from transient kinetic experiments with physico-chemical parameters. Plots of the logarithms of the rate constants of hydroxylation (k_{OH}) *versus* DFT calculated values of *(A)* free energy of deprotonation (ΔG_{De}, R = 0.95), *(B)* free energy of -OH transfer (ΔG_{OH}, R = 0.97), *(C)* free energy of overall hydroxylation ($\Delta G_{OH,Overall}$, R = 0.95), *(D)* energy gap between the highest occupied molecular orbital (E_{HOMO}) of phenols (in phenolate formed) and the lowest unoccupied molecular orbital (E_{LUMO}) of C4a-hydroperoxy-FAD (R = 0.92), and *(E)* bond length of C_1-O (R = 0.96).
